# State‐of‐the‐Art Strategies for Circular RNA in Cancers: Opportunity and Challenge

**DOI:** 10.1002/mco2.70608

**Published:** 2026-02-08

**Authors:** Zehao Ding, Zai Luo, Liao Zhang, Shaopeng Zhang, Renchao Zhang, Zhengjun Qiu, Chen Huang

**Affiliations:** ^1^ Department of Gastrointestinal Surgery The Affiliated Chuzhou Hospital of Anhui Medical University AnHui China; ^2^ Institute of Gastrointestinal Tumor Research The First People's Hospital of Chuzhou AnHui China; ^3^ Department of Gastrointestinal Surgery Shanghai General Hospital，Shanghai Jiao Tong University School of Medicine Shanghai China; ^4^ Institute of Gastrointestinal Tumor Research Shanghai General Hospital,Shanghai Jiao Tong University School of Medicine Shanghai China

**Keywords:** circular RNA, biomarkers, immune evasion, metabolic reprogramming, therapeutic targeting, tumorigenesis

## Abstract

Circular RNAs (circRNAs) are characterized by their covalently closed structure, remarkable stability, and precise spatiotemporal regulation, evolving from once‐overlooked transcriptional byproducts to pivotal molecular regulators. In addition to their well‐established function as microRNA sponges, circRNAs serve as protein scaffolds, transcriptional modulators, and even templates for functional peptide synthesis. This review synthesizes recent breakthroughs across the entire circRNA life cycle, encompassing biogenesis, degradation, nucleocytoplasmic transport, and extracellular vesicle‐mediated secretion, while systematically analyzing their multifaceted involvement in tumorigenesis, immune evasion, metastatic dissemination, programmed cell death, and tumor–microbiome crosstalk. We highlight their exceptional potential as liquid biopsy biomarkers and critically assess translational applications in circRNA‐based vaccines, targeted delivery platforms, and engineered cell therapies like CAR‐T. Emerging artificial intelligence approaches that accelerate circRNA discovery, functional characterization, and therapeutic design are also discussed. Addressing current challenges in standardization and delivery methodologies, we propose future directions for incorporating circRNAs and their encoded proteins into precision oncology and next‐generation immunotherapies. Together, these advances position circRNAs as a transformative paradigm with the potential to revolutionize cancer diagnostics, targeted therapeutics, and RNA vaccine development.

## Graphical abstract

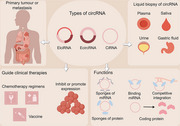

## 1 Introduction

CircRNAs constitute a unique class of noncoding RNAs (ncRNAs), distinguished by their circular structure formed through covalently closed loops of multiple nucleotides [1]. The first observation of circRNA was made in an RNA virus in 1976 [2]. Initially, circRNA was regarded as a result of aberrant splicing, a perspective largely shaped by technological limitations and other factors. However, with the ongoing advancements in sequencing technology and bioinformatics, it has become evident that circRNA constitutes an abundant and evolutionarily conserved class of RNA, demonstrating widespread and intricate tissue‐, cell type‐, or stage‐specific expression patterns [3, 4]. Unlike linear RNAs, circRNAs undergo noncanonical splicing to form a covalently closed loop structure without free 3′ or 5′ ends. This unique structure makes them less susceptible to degradation by exonucleases and thus more stable than linear RNAs [5]. In recent years, numerous studies have confirmed that circRNAs possess critical biological functions and play a vital role in the onset and progression of diseases. For instance, specific subsets of circRNAs have been shown to promote tumor proliferation, migration, and metabolic reprogramming [6, 7, 8, 9]. Moreover, translation‐competent circRNAs encode functional proteins that regulate oncogenic signaling pathways [10, 11]. Recent advances have further elucidated their roles in mediating therapeutic resistance and highlighted their potential as engineered circRNA vaccines for emerging immunotherapeutic strategies [12, 13, 14, 15, 16]. Emerging evidence from various oncological studies indicates that certain circRNAs show significant potential as novel tumor biomarkers, potentially outperforming traditional markers like CEA [17, 18, 19].

Global cancer epidemiology continues to be a critical research priority. According to the latest World Health Organization assessment, there were 19.96 million new cancer cases and 9.74 million cancer‐related deaths worldwide in 2022 [20]. In 2013, two groundbreaking studies published consecutively in *Nature* clarified the molecular mechanisms by which circRNAs function as competitive endogenous RNAs (ceRNAs). These studies provided the first definitive evidence that circRNAs regulate miRNA activity through their sponging effects [21, 22]. These landmark discoveries reshaped our understanding of circRNAs, elevating them from perceived “splicing artifacts” to a diverse category of endogenous regulatory RNA molecules. As circRNA research has advanced, their multifaceted biological roles have been increasingly elucidated. In 2017, two studies published in *Molecular Cell* provided the first definitive evidence that endogenous circRNAs have protein‐coding potential in vivo [23, 24]. Specifically, circ‐ZNF609, originating from myoblasts, was demonstrated to undergo cap‐independent translation, yielding a functional protein [23]. This groundbreaking discovery not only challenges the conventional understanding of circRNAs but also opens new avenues in ncRNA and proteomics research. In 2022, Wei and colleagues achieved a significant breakthrough by developing a circRNA‐based vaccine effective against SARS‐CoV‐2 and its variants of concern. This innovation not only provided a promising new vaccine candidate for COVID‐19 but also established a groundbreaking technological platform. The success of this approach has since facilitated rapid advancements in circRNA‐based therapeutics and vaccine development, with applications extending to oncology research [25].

**FIGURE 1:**
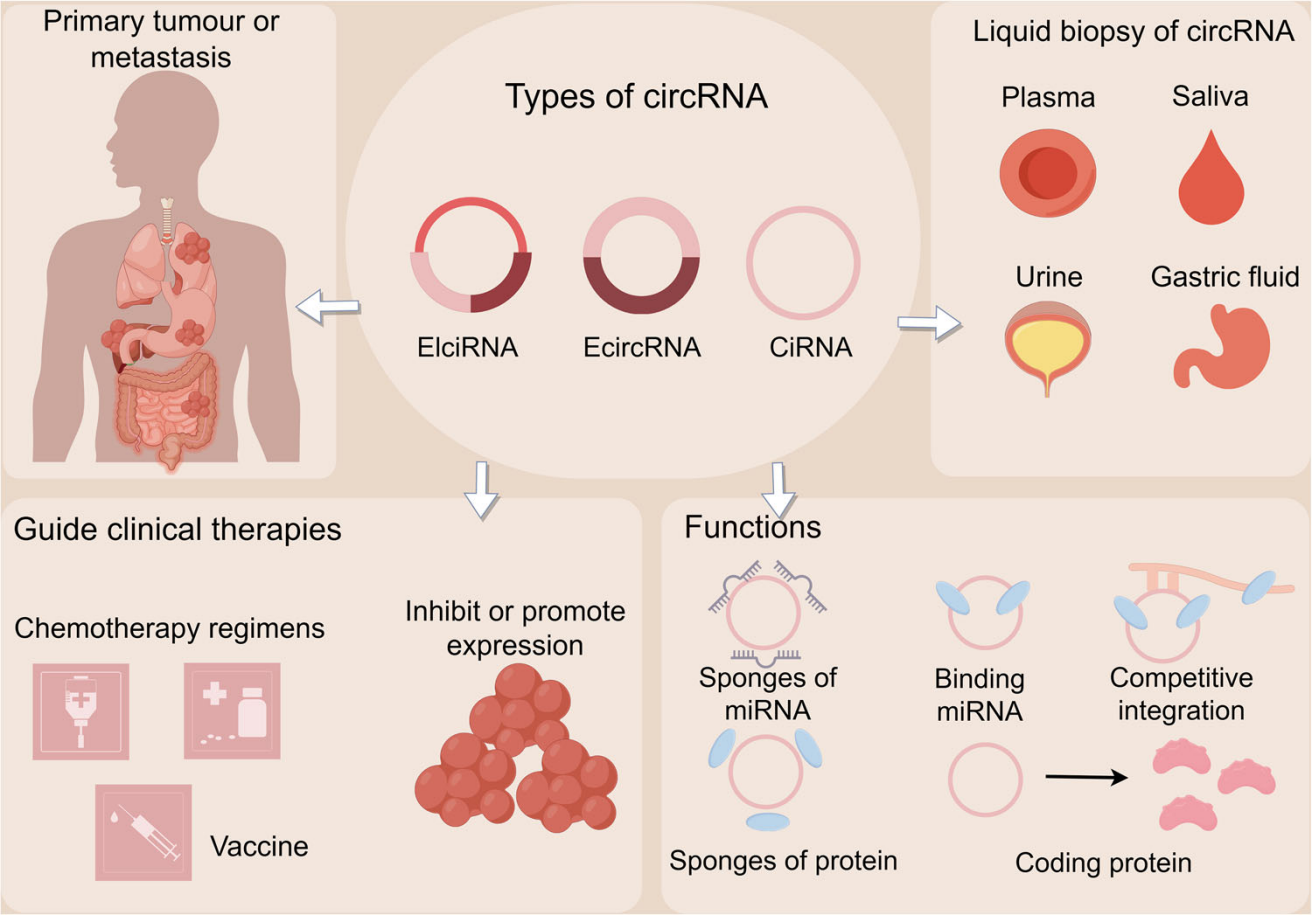
Overview of circular RNA in tumors. Various types of circRNAs can be detected through liquid biopsies from plasma, saliva, urine, and gastric fluid, providing insights into the status of both primary and metastatic tumors. By functioning as sponges for miRNAs and proteins, engaging in competitive interactions, and encoding peptides, circRNAs have the potential to guide clinical therapies, including chemotherapy regimens, vaccines, and strategies to modulate circRNA expression.

This review offers a comprehensive overview of the evolving field of circRNAs, covering their biogenesis, classification, degradation, and regulatory factors. We delve into their functional mechanisms, emphasizing their roles in tumorigenesis and their emerging protein‐coding potential. Additionally, we discuss the clinical potential of circRNAs, particularly as biomarkers for early cancer detection and as novel therapeutic targets (Figure 1). Last, we highlight the transformative impact of artificial intelligence on circRNA research and address the challenges in translating these findings into clinical practice.

## 2 Biosynthesis of CircRNA

**FIGURE 2:**
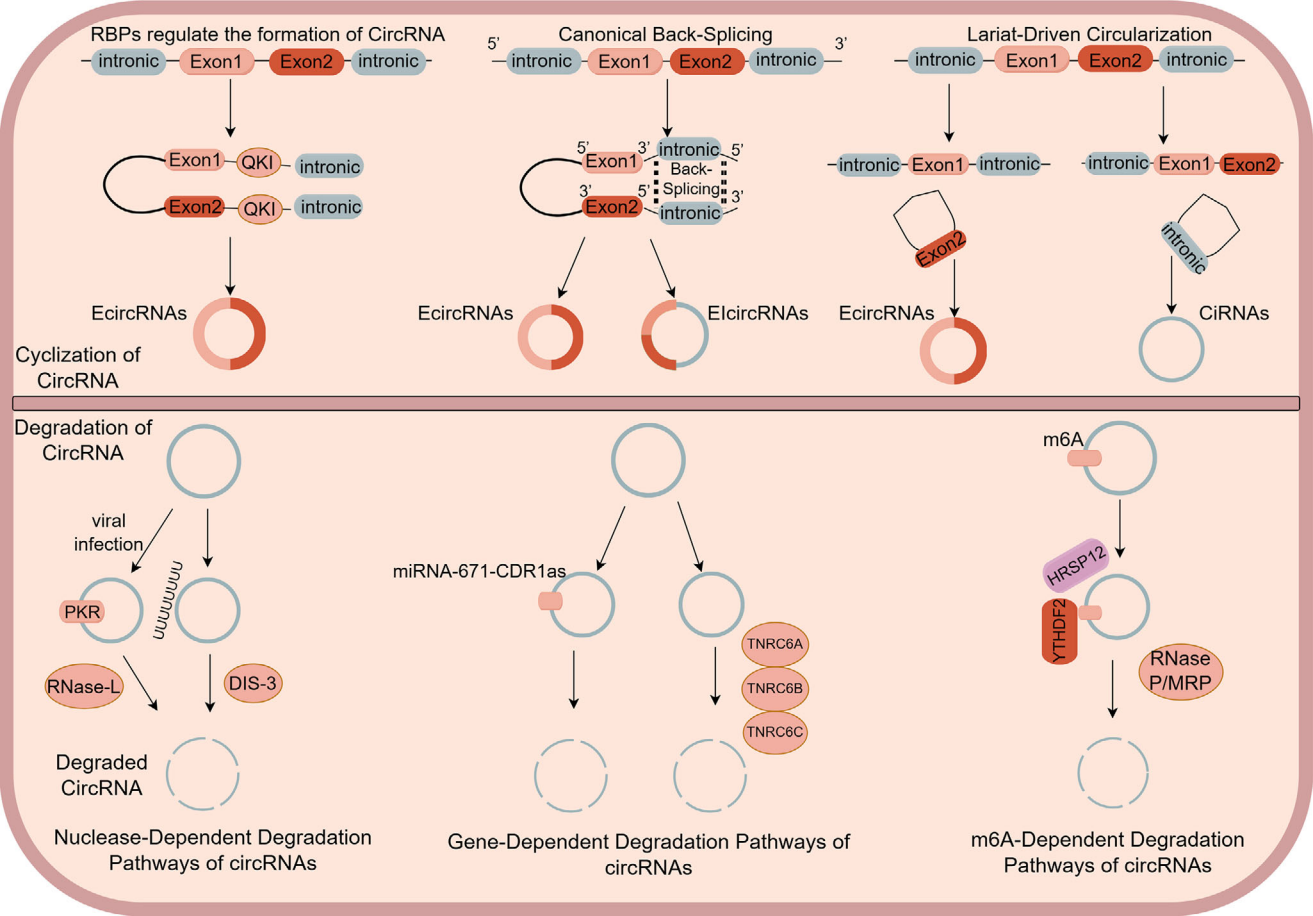
The mechanisms and processes of cyclization and degradation of circRNA. The upper panel illustrates the circularization of precursor mRNAs into circRNAs through three mechanisms: RBP‐mediated exon circularization, canonical back‐splicing of flanking introns, and lariat‐driven circularization. The lower panel summarizes the major degradation pathways of circRNAs, including: (1) nuclease‐dependent pathways activated by viral infection; (2) gene‐dependent degradation, such as miR‐671‐directed CDR1as decay mediated by TNRC6 family proteins; and (3) N^6^‐methyladenosine (m^6^A)‐dependent degradation.

The biogenesis, degradation, and intercellular communication of circRNAs exhibit remarkable mechanistic diversity in humans. Different subtypes of circRNAs undergo different modes of formation and turnover [26]. A comprehensive understanding of these processes is essential for elucidating the context‐dependent functions of circRNAs across various diseases. Therefore, we summarize the current knowledge on the major pathways governing circRNA circularization, degradation, as well as their nuclear export and extracellular transport (Figure 2).

### 2.1 Circularization Mechanisms of CircRNA

#### 2.1.1 Canonical Back‐Splicing

Similar to linear RNAs, circRNAs are also derived from precursor RNAs and transcribed by RNA polymerase II. However, their splicing process is distinct: the pre‐mRNA forms a loop through covalent bonding between the 5′ splice donor site downstream of an exon and the 3′ splice acceptor site upstream [27]. This loop then undergoes intron removal by cleavage to produce exonic circRNAs (EcircRNAs). During this reverse splicing process, some introns may remain uncleaved, resulting in the formation of exon–intron circRNAs (EIcircRNAs) [28].

#### 2.1.2 Lariat‐Driven Circularization

Unlike the canonical back‐splicing mechanism, lariat‐driven circularization begins with exon‐skipping events during pre‐mRNA splicing. In this process, flanking introns are ligated into circular structures, while the skipped exons form m^7^G‐capped lariat intermediates. Subsequent back‐splicing then produces mature EcircRNAs. Multiple studies have shown that SF3B1 impairs exon recognition, thereby promoting intronic EIcircRNA biogenesis through exon‐skipping‐dependent circularization [29, 30]. The lariat‐driven formation of intronic circRNAs (CiRNAs) fundamentally differs from that of EcircRNAs. During canonical splicing, intronic lariats are typically degraded. However, if they contain a 7‐nucleotide GU‐rich motif at the 5′ splice site and an 11‐nucleotide C‐rich sequence near the 3′ end, these lariats evade debranching in standard linear splicing and instead fold into circular conformations via back‐splicing (Figure 2) [31, 32].

#### 2.1.3 RNA‐Binding Proteins‐Dependent Cyclization Mechanism

Certain RNA‐binding proteins (RBPs) enhance circRNA biogenesis by specifically recognizing motifs within the flanking introns of pre‐mRNAs. Through either dimerization induction or complementary sequence bridging, these RBPs promote the spatial approximation of upstream and downstream back‐splice sites [33]. This structural rearrangement establishes an optimal configuration for the spliceosome to catalyze back‐splicing and generate circRNAs (Figure 2) [34]. The initial dimerization event allows RBPs to bridge adjacent exons, as illustrated by the Quaking protein, which forms homodimers through its C‐terminal (C‐term) domain, effectively clamping down on target motifs in a spatially constrained manner [35]. Furthermore, the FUS (fused in sarcoma) protein binds to the intronic regions flanking back‐splicing junctions (BSJs), dynamically modulating circRNA biogenesis. It exhibits context‐dependent dual functionality, acting as both a splicing activator and repressor [36]. Additionally, certain RBPs compete for binding to splice sites or regulatory elements, thereby suppressing the formation of linear mRNA and indirectly enhancing back‐splicing. For example, Kadener et al. reported that the muscleblind (MBL) protein binds to sequences surrounding the third intron of its own pre‐mRNA, promoting the production of the circular transcript circMbl while simultaneously inhibiting the canonical splicing of the corresponding linear isoform [37].

### 2.2 Classification of CircRNA

**FIGURE 3:**
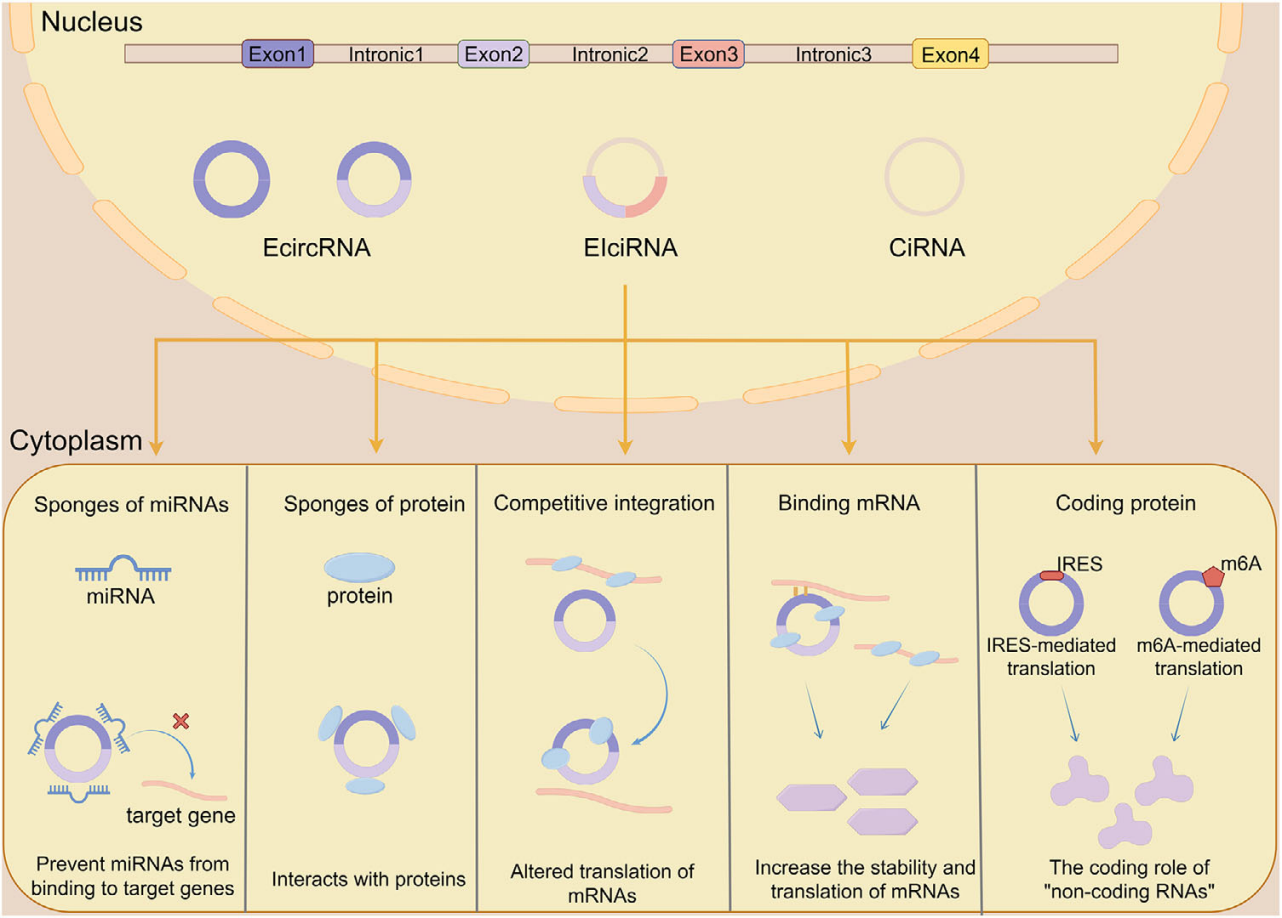
Classification and function of circular RNA. In the nucleus, precursor mRNAs undergo back‐splicing to generate EcircRNAs, exon–EIcircRNAs, and ciRNAs. Upon export to the cytoplasm, circRNAs engage in various regulatory processes, including: (1) functioning as molecular sponges for miRNAs and RNA‐binding proteins; (2) competitively modulating mRNA translation; (3) enhancing the stability and translational efficiency of target transcripts; and (4) serving as templates for protein or micropeptide synthesis through IRES‐ or m6A‐mediated cap‐independent translation.

Most of the literature categorizes circRNAs into three main types: EcircRNAs, EIcircRNAs, and CiRNAs [38]. Recent studies have demonstrated that circRNAs encompass not only antisense circRNAs but also intergenic circRNAs [39]. The majority of identified circRNAs are exon circRNAs and intronic circRNAs. Although the splicing of circRNAs primarily occurs in the nucleus, EcircRNAs are predominantly exported to the cytoplasm, whereas CiRNAs remain sequestered in the nucleus (Figure 3) [27, 39].

### 2.3 Mechanisms of CircRNA Degradation

#### 2.3.1 Nuclease‐Dependent Degradation Pathways of CircRNAs

Previous studies have established that RNase L, an interferon‐induced endoribonuclease, mediates comprehensive degradation of circRNAs during viral infection [40]. Clinical observations have revealed marked decreases in circRNA levels in patients with systemic lupus erythematosus and chronic inflammatory skin disorders [40]. Under normal physiological conditions, circRNAs form intramolecular double‐stranded structures (16–26 bp) that interact with and inhibit dsRNA‐activated protein kinase (PKR), thereby preserving immune equilibrium. During viral infection, viral dsRNA stimulates 2′–5′‐oligoadenylate synthetases to generate 2–5A, which activates RNase L's endonucleolytic function and initiates circRNA breakdown [41]. Simultaneously, PKR‐mediated phosphorylation of eIF2α suppresses overall protein synthesis to inhibit viral proliferation. Importantly, this degradation pathway, which is specifically induced during viral infection, does not fully explain circRNA turnover under physiological circumstances [41]. A recent study by Chen et al. uncovered a novel degradation pathway mediated by DIS3 (chromosome transmission fidelity protein 3), a bifunctional endo/exoribonuclease that maintains circRNA homeostasis in noninfected cells [42].

DIS3 encodes a highly conserved ribonuclease with dual catalytic activities: 3′–5′ exoribonuclease activity mediated by its RNB domain and endoribonuclease activity through the PIN (PilT N‐terminus) domain [43]. Genetic studies demonstrate that DIS3 depletion leads to a significant upregulation of over 60% of circRNA, while showing minimal impact on their linear counterparts [44]. This DIS3‐mediated circRNA degradation is evolutionarily conserved, occurs in the cytoplasm, and strictly depends strictly on DIS3's endonucleolytic activity, yet remains independent of the RNA exosome complex. Additionally, sequence enrichment analysis indicates that DIS3 preferentially targets circRNAs containing U‐rich motifs, suggesting sequence‐specific recognition [45].

#### 2.3.2 Gene‐Dependent Degradation Pathways of CircRNAs

In contrast to enzyme‐mediated degradation pathways, multiple genes have been identified as participating in circRNA decay through distinct mechanisms [46, 47]. Ago2, a ubiquitously expressed member of the Argonaute protein family, plays a key role in this process. CDR1as, a functionally important circRNA containing over 60 miR‐7 binding sites with critical implications in various pathologies, demonstrates near‐perfect complementarity to miRNA‐671 [48]. This structural feature facilitates experimental confirmation of Ago2‐mediated cleavage and degradation of CDR1as upon recognition of the miRNA‐671–CDR1as complex (Figure 2). Additionally, GW182 has been shown to participate in circRNA degradation. RNAi library screening in Drosophila DL1 and S2 cells revealed significant enrichment of specific circRNAs following GW182 depletion. Systematic genetic screens have uncovered protein‐coding genes that functionally regulate circRNA degradation pathways. Genome‐wide RNAi screening in Drosophila DL1 and S2 cells identified GW182 as a pivotal regulator of circRNA turnover. GW182 depletion markedly increased the abundance of circular isoforms originating from the dati and laccase2 transcripts. Structural analysis demonstrated that GW182 possesses six functionally distinct domains: an Ago‐binding domain, ubiquitin‐associated domain (UBA), glutamine‐rich domain (Q‐rich), middle region (Mid), RNA‐recognition motif, and C‐term region. Systematic deletion mutagenesis revealed that specifically disrupting the Mid domain eliminated the circRNA destabilization effect, highlighting its essential role in circRNA degradation. In humans, GW182 orthologs consist of three genes: TNRC6A, TNRC6B, and TNRC6C. Knockdown of each ortholog consistently increased circRNA accumulation, demonstrating their conserved function in circRNA decay pathways [48].

#### 2.3.3 N^6^‐Methyladenosine‐Dependent Degradation Pathways of CircRNAs

N^6^‐methyladenosine (m^6^A) is a prevalent internal modification found in eukaryotic mRNAs and ncRNAs. Its canonical function in circRNAs involves regulating RNA stability through the recruitment of specific reader proteins and degradation complexes [49]. YTHDF2 is an evolutionarily conserved RBP characterized by a canonical YTH (YT521‐B homology) domain that specifically recognizes and binds to m^6^A‐modified RNA [50]. YTHDF2 recognizes m^6^A modifications and recruits the adaptor protein HRSP12, along with the RNase P/MRP endonuclease complex, to specific sites on circRNAs, where HRSP12 binds upstream of the YTHDF2 binding site and RNase P/MRP cleaves at downstream positions (Figure 2). This coordinated recruitment results in precise circRNA cleavage and subsequent rapid degradation [51, 52].

### 2.4 Nuclear Export Mechanisms of CircRNAs

CircRNAs are primarily generated within the nucleus through backsplicing. However, their functional roles, such as serving as miRNA sponges, interacting with proteins, or undergoing translation, require precise subcellular localization, mainly in the cytoplasm, although a subset remains in the nuclear. Therefore, the nuclear export processes of circRNAs are tightly regulated.

**FIGURE 4:**
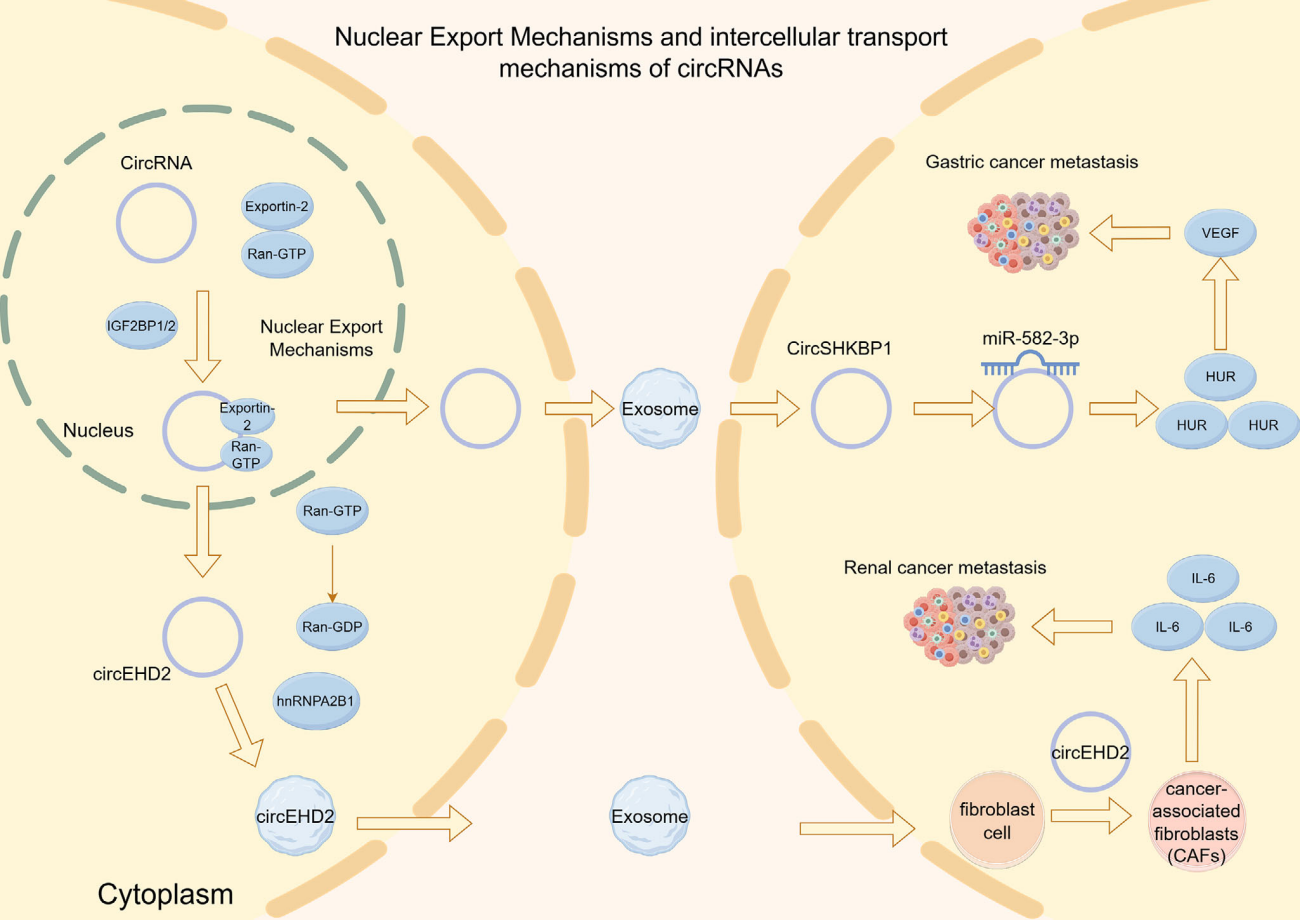
Intercellular communication and function of circular RNA. CircRNAs are exported from the nucleus to the cytoplasm via pathways involving exportins and Ran GTPase. They are subsequently packaged into exosomes and transported to neighboring cells. This exosome‐mediated intercellular communication enables circRNAs to reshape the tumor microenvironment and promote cancer metastasis.

Ran‐GTP, a small GTPase, forms an intracellular concentration gradient that provides directional cues for various nucleocytoplasmic transport processes [53]. As a nuclear export receptor, Exportin‐2 (XPO2) mediates the translocation of RNA molecules from the nucleus to the cytoplasm. Notably, XPO2 serves as the sole export receptor for circRNA nuclear export, though it does not directly bind circRNA. This process relies critically on the adaptor proteins IGF2BP1 and/or IGF2BP2, which are mRNA localization‐associated factors. These adaptors demonstrate increased circRNA binding affinity in the presence of Ran‐GTP. Thus, circRNAs are exported via a Ran‐GTP‐dependent pathway, where XPO2 functions as the export receptor while IGF2BP1 (or IGF2BP2) physically links the circRNA cargo to the XPO2/Ran‐GTP complex. Upon cytoplasmic hydrolysis of Ran‐GTP to Ran‐GDP, the circRNA is subsequently released (Figure 4) [54].

### 2.5 The Intercellular Transport Mechanisms of CircRNA

Emerging evidence suggests that circRNAs not only function intracellularly but also participate in intercellular communication through extracellular secretion and uptake by neighboring or distant cells, playing pivotal roles in developmental, immune, and oncogenic processes [55, 56]. This intercellular transport is primarily mediated by extracellular vesicles (EVs).

Exosomes, a major EV subtype ranging from 40 to 160 nm in diameter, are secreted by various cell types and serve as vehicles for transferring lipids, proteins, DNA, and ncRNAs [57]. Following uptake by recipient cells, exosome‐derived circRNAs can exert regulatory functions, most notably through their miRNA sponge activity. By competitively binding to miRNAs, these circRNAs prevent miRNA interaction with target gene 3′‐untranslated regions (UTRs), effectively modulating downstream signaling pathways implicated in cancer progression and metastasis (Figure 4) [58, 59, 60]. For instance, Xie et al. revealed that exosomal circSHKBP1 promotes gastric cancer metastasis by sponging miR‐582‐3p, which subsequently elevates HUR expression. This upregulation stimulates VEGF secretion and angiogenesis in gastric cancer tissues [61]. Similarly, Liu et al. discovered that circEHD2, which is highly expressed in renal cancer tissues and metastatic patient serum exosomes, is selectively packaged into exosomes through hnRNPA2B1 binding. Following exosomal transfer to fibroblasts, circEHD2 induces their transformation into cancer‐associated fibroblasts (CAFs). These activated CAFs then secrete IL‐6, creating a prometastatic microenvironment that drives renal cancer progression [62] (Figure 4).

## 3 Characteristics of CircRNAs

The key characteristics of circRNAs encompass their remarkable structural stability, distinct tissue‐ and disease‐specific expression, and significant spatiotemporal heterogeneity across various cell types, tumor regions, and microenvironmental components. These attributes underpin the functional roles of circRNAs, influencing their expression patterns and regulatory functions in cancer and other diseases [63]. Our review integrates these three fundamental characteristics and elucidates the underlying principles that support their potential as biomarkers and their broader clinical applications.

### 3.1 High Stability

CircRNAs are generated through the backsplicing of pre‐mRNA, resulting in covalently closed circular structures that lack the 5′ cap and 3′ poly(A) tail found in linear RNAs. This unique topology enables circRNAs to evade deadenylation, decapping, and exonucleolytic degradation. Quantitative studies in mammary gland cells have revealed a median circRNA half‐life of 18.8 to 23.7 h, in stark contrast to the 4.0–7.4‐h half‐life observed for their homologous linear RNAs [64]. Furthermore, comparative half‐life analysis has shown that m^6^A‐modified circNSUN2 exhibits a stability of over 24 h in colorectal cancer cells, sharply contrasting with the approximately 4‐h half‐life of its linear transcript counterpart [65]. Consequently, circRNAs are considered to be stably preserved in bodily fluids such as plasma, exosomes, and urine. The noninvasive detection of circRNA expression changes in these fluids allows for effective monitoring of dynamic circRNA alterations in cancer patients. Through genome‐wide screening, Zhang et al. identified a biomarker panel consisting of five circRNAs, which can be used for the early noninvasive detection of pancreatic ductal adenocarcinoma (PDAC). Notably, when combined with CA19‐9, the diagnostic performance is significantly enhanced [66].

### 3.2 Tissue Specificity

Extensive studies have confirmed that circRNAs are ubiquitously expressed across eukaryotic tissues, with notable enrichment in mammalian brain and skeletal muscle [21, 67, 68]. Tumor‐specific circRNA expression exhibits significant heterogeneity: for instance, circFAM53B is specifically enriched in breast cancer tissues but undetectable in normal mammary glands, a pattern also observed in melanoma [69]. CircE7 is the only identified HPV‐encoded circRNA, exclusively expressed in HPV‐positive head and neck squamous cell carcinoma (HNSCC) [70]. Additionally, circZKSCAN1 is highly expressed in normal liver, where it maintains cellular homeostasis, but is significantly downregulated in hepatocellular carcinoma (HCC). Interestingly, this circRNA is significantly upregulated in tumor tissues such as colorectal and lung ADCs [71]. A study on cervical cancer identified 215 circRNAs that were upregulated in SCC but downregulated in ADC, with 50 showing inverse regulation patterns [72]. Moreover, numerous circRNAs exhibit dysregulated expression patterns across various cancer types. For example, CircHIPK3 (hsa_circ_0000284), a circular RNA (circRNA) derived from the exons of the HIPK3 gene, is widely expressed in diverse tissues and cell types. It is significantly upregulated in several malignancies, including gastric, colorectal, and breast cancers, but notably downregulated in bladder cancer [73]. Overall, circRNAs display distinct tissue‐specific expression patterns in vivo. Comprehensive sequencing analyses of various tumor tissues enable researchers to elucidate the complex regulatory functions of specific circRNAs in tumor initiation and progression, as well as their dynamic roles across diverse biological contexts [74].

### 3.3 Spatiotemporal Heterogeneity

The exceptional stability and tissue specificity of circRNAs provide a solid foundation for functional studies, while their expression patterns exhibit spatiotemporal dynamics across various biological contexts.

Multiple tumor studies have confirmed the spatiotemporal heterogeneity of circRNAs. For instance, in gliomas, the most prevalent primary malignant brain tumors with a poor prognosis, circRNA sequencing revealed a NEIL3‐derived circRNA that is upregulated in tumor tissues and shows expression levels positively correlated with pathological grades (I–IV) [75]. A groundbreaking study published in *Nature Communications* utilized spatial analysis technologies to investigate the distribution of CDR1as in colon cancer tissues. Surprisingly, the researchers discovered that ciRS‐7 was not localized within cancer cells but was highly expressed in the stromal cells of the tumor microenvironment (TME). This finding challenges the conventional view that CDR1as is an endogenous molecule of cancer cells and underscores the spatial heterogeneity of circRNAs across different tumor regions [76]. Moreover, circRNA profiles exhibit significant functional heterogeneity between tumor cells and the TME. In tumor cells, circRNAs primarily regulate malignant behaviors such as proliferation, invasion, metastasis, metabolic reprogramming, epithelial–mesenchymal transition (EMT), and drug resistance. By contrast, circRNAs within TME cells mainly participate in exosome‐mediated intercellular communication, affecting processes like immunosuppression, fibrosis, and angiogenesis [77]. Shi et al. reported that exosomal circUHRF1 in liver cancer suppresses natural killer (NK) cell function and induces NK cell exhaustion by inhibiting miR‐449c‐5p, thereby upregulating TIM‐3 expression [78]. Similarly, Kang et al. demonstrated that exosomes derived from CRC cells promote vascular endothelial cell migration and tube formation by inducing filopodia formation and endothelial tip cell orientation. Silencing circTUBGCP4 in CRC cell‐derived exosomes (CRC‐CDEs) effectively inhibited endothelial cell migration, tube formation, tip cell formation, and CRC metastasis [79].

## 4 Functional Mechanisms of CircRNAs

The diverse regulatory mechanisms of circRNAs form a crucial foundation for their roles in cancer research and clinical applications, allowing them to exert their biological functions through multiple pathways [26]. Our review summarizes their primary functional mechanisms, which include serving as ceRNAs to modulate gene expression, interacting with RBPs to influence transcriptional programs and protein activity, and generating biologically active peptides through noncanonical translation (Figure 3).

### 4.1 Competing Endogenous RNA

The role of circRNAs has garnered significant attention. miRNAs are a class of endogenous small RNAs, typically 20–24 nucleotides in length. Multiple miRNAs can coregulate the expression of individual genes, thereby fine‐tuning gene expression and participating in various physiological activities [80]. Numerous studies have demonstrated that circRNAs function as molecular sponges by competitively binding to miRNAs [81, 82, 83]. This interaction blocks miRNA‐target mRNA binding, thereby relieving the suppression of target genes. Consequently, circRNAs precisely modulate downstream signaling pathways. This intricate circRNA–miRNA–mRNA regulatory axis exerts profound biological effects on tumorigenesis and progression across diverse cancer types [84]. For instance, circ‐CDYL accelerates early‐stage HCC progression by sponging miR‐892a and miR‐328‐3p, leading to the upregulation of hepatoma‐derived growth factor (HDGF) and hypoxia‐inducible factor asparaginyl hydroxylase (HIF1AN). This mechanism activates the HDGF‐mediated PI3K–AKT–mTORC1/β‐catenin pathway while suppressing HIF1AN‐regulated NOTCH2 signaling [85]. Additionally, Mou et al. identified significantly downregulated circRNF216 expression in colorectal cancer tissues and cells. Functioning as a ceRNA, circRNF216 sequesters miR‐576‐5p, thereby alleviating its suppression of the target gene ZC3H12C and reducing N‐cadherin levels [86]. Furthermore, circRNF216 enhances CD8+ T cell infiltration by upregulating ZC3H12C, ultimately inhibiting colorectal cancer progression. Downregulation of circCCDC9 was observed in gastric cancer cells, and functional analysis demonstrated its direct binding to miR‐6792‐3p, which alleviates the suppression of the tumor suppressor gene CAV1 [87].

### 4.2 CircRNA–RBP Interaction Patterns

RBPs constitute a class of proteins that specifically interact with RNA molecules. By recognizing distinct RNA sequences or structures, RBPs regulate the entire RNA life cycle, including posttranscriptional processes, and their functional dysregulation is closely associated with various disease pathologies [88, 89].

First, circRNAs can specifically bind RBPs, thereby influencing their interactions with other proteins. For example, circ0006646, a circRNA associated with HCC and poor prognosis, binds nucleolin (NCL) and disrupts its interaction with the E3 ubiquitin ligase TRIM21. This suppression of K48‐linked ubiquitination and degradation stabilizes the oncogenic protein NCL. Accumulated NCL inhibits p53 translation in vivo, ultimately promoting cancer cell metastasis [90, 91]. In laryngeal SCC, circMTCL1 directly binds the RBP C1QBP, inhibiting its ubiquitination‐mediated degradation. This complex coactivates Wnt/β‐catenin signaling, driving disease progression in vivo [92]. Second, circRNAs can interact with specific proteins to dysregulate their native functions or elicit novel biological effects. CircHuR suppresses the proliferation, invasion, and metastasis of gastric cancer by inhibiting HuR transcription through blocking the binding of the transcription factor CNBP to the HuR gene promoter [93]. Circ‐hnRNPU, an exon‐derived circRNA from heterogeneous nuclear ribonucleoprotein U (hnRNPU), physically interacts with the non‐POU domain‐containing octamer‐binding protein (NONO). This interaction induces cytoplasmic retention of NONO, thereby suppressing tumor progression through downregulation of glycosyltransferases. The underlying mechanism involves inhibiting both nuclear NONO‐mediated c‐Myc transcriptional activation and cytoplasmic NONO‐promoted mRNA stabilization [94]. Third, circRNAs can bind specific cis‐regulatory elements to modulate transcription factors or epigenetic modifications, thereby altering gene expression. For instance, circRHOT1 recruits and anchors TIP60 (a histone acetyltransferase) to the NR2F6 promoter region, activating its transcription and consequently promoting HCC proliferation and metastasis [95]. Additionally, circMRPS35 specifically recruits KAT7 to the FOXO1/FOXO3a promoters, inducing H4K5 acetylation to activate transcription. This process orchestrates a tumor‐suppressive transcriptional network involving p21, p27, and E‐cadherin, thereby inhibiting gastric cancer proliferation and metastasis [96].

### 4.3 CircRNA Encode Proteins

In addition to their previously recognized functions, recent studies have revealed that some circRNAs can undergo translation. Advances in bioinformatics platforms and next‐generation sequencing technologies have enabled the identification of numerous circRNAs containing open reading frames (ORFs) capable of encoding proteins [97]. Due to the absence of 5′ caps and 3′ termini, circRNA translation is facilitated through cap‐independent mechanisms. Internal ribosome entry sites (IRES), located within the 5′ untranslated regions of mRNAs, enable translation initiation in the absence of canonical initiation factors, either partially or completely [96]. The IRES‐dependent translation mechanism is widespread among circRNAs. CircRNAs can initiate translation by directly recruiting ribosomes through the insertion of a synthetic IRES upstream of the start codon. Fan et al. discovered that numerous short IRES‐like elements are significantly enriched in endogenous circRNAs, serving as trans‐acting factors that promote cap‐independent translation [98]. Another mechanism of circRNA translation is mediated by m^6^A, the most prevalent internal RNA modification in eukaryotes. Yang et al. found that a single m^6^A site effectively recruits the 43S complex to initiate translation, aided by the YTH domain family protein 3 (YTHDF3) and eIF4G2. The translation is inhibited by the m^6^A demethylase FTO but enhanced by the adenosine methyltransferases METTL3/14 [99]. Pan et al. identified that circ‐YAP is significantly upregulated in colorectal cancer with liver metastasis and is associated with poor prognosis. This circRNA encodes a novel 220‐amino acid truncated protein, termed YAP‐220aa, which competitively binds LATS1, leading to YAP dephosphorylation and nuclear translocation. Consequently, this process activates prometastatic genes [100]. Additionally, circCAPG encodes a 171‐amino acid protein that binds to the kinase STK38, disrupting its interaction with the E3 ubiquitin ligase SMURF1. This disruption suppresses MEKK2 ubiquitination and degradation, leading to sustained activation of the MEK/ERK signaling pathway, thereby promoting proliferation and metastasis in triple‐negative breast cancer (TNBC) cells [101].

## 5 Biological Function of CircRNAs in Tumors

Emerging evidence indicates that circRNAs play pivotal regulatory roles in cancer by modulating key biological processes, including tumor cell proliferation, immune evasion, invasion, and metastasis, and various forms of cell death. Additionally, circRNAs are intricately involved in metabolic reprogramming, cellular senescence, and epigenetic regulation. They also influence the remodeling of the TME through interactions with the microbiota [77]. By summarizing these mechanisms, we can elucidate how circRNAs coordinate tumor initiation, progression, and metastasis across multiple biological levels (Table 1).

**TABLE 1:** CircRNA as tumor biomarker and its mechanism of action in tumors.

CircRNA	Cancer type	Sample type	Expression	Functional mechanism	Clinical relevance	References
circSHKBP1	Gastric cancer	Tissue/exosome	Upregulated	Promotes angiogenesis and metastasis via miR‐582‐3p/HUR/VEGF	Diagnostic/prognostic	[61]
circEHD2	Renal cancer	Serum exosome	Upregulated	Activates CAF transition via hnRNPA2B1‐mediated IL‐6 secretion	Prognostic	[62]
circUHRF1	Hepatocellular carcinoma	Exosome	Upregulated	Sponges miR‐449c‐5p → TIM‐3 ↑ → NK cell exhaustion	Immune evasion/prognostic	[78]
circTUBGCP4	Colorectal cancer	Exosome	Upregulated	Promotes endothelial migration and tube formation	Prognostic	[79]
circRNF216	Colorectal cancer	Tissue	Downregulated	Sponges miR‐576‐5p → ↑ZC3H12C → ↑CD8^+^ T‐cell infiltration	Diagnostic/tumor suppression	[86]
circp53 (hsa_circp53_0041947)	Multiple myeloma	Tissue	Downregulated	Encodes p53‐209aa → activates mitochondrial apoptosis	Diagnostic/therapeutic	[102]
circSATB1	Colorectal cancer	Tissue	Upregulated	RNF25/FKBP8/mTOR/EMT axis → metastasis	Prognostic	[103]
circFAM13B	Nasopharyngeal carcinoma	Tissue	Downregulated	Inhibits lymphangiogenesis/↑5‐year survival	Prognostic	[104]
circTFRC	Gastric cancer	Tissue	Upregulated	Recruits ELAVL1 → stabilizes SCD1 mRNA → ferroptosis resistance	Therapeutic target	[105]
circMAT2B	Hepatocellular carcinoma	Tissue	Upregulated	miR‐338‐3p/PKM2 axis → glycolysis ↑	Metabolic reprogramming/prognostic	[106]
circACC1	Colorectal cancer	Tissue	Upregulated	Forms complex with AMPK β/γ subunits → fatty acid metabolism ↑	Prognostic	[107]
circ_0000003	Tongue SCC	Tissue	Upregulated	miR‐330‐3p/GLS axis → ↑α‐KG, ↑ATP → proliferation ↑	Prognostic	[108]
circ_0000808	NSCLC	Tissue	Upregulated	miR‐1827/SLC1A5 axis → ↑glutamine metabolism	Prognostic	[109]
circCDYL	Breast cancer	Tissue	Upregulated	Sponges miR‐1275 → ↑ATG7/ULK1 → promotes autophagy	Prognostic	[110]
circHIPK2	Colorectal cancer	Tissue	Upregulated	FUS–EIF4A3 complex → ↑TAZ translation	Early diagnostic biomarker	[111]

### 5.1 The Role of CircRNAs in Tumor Growth and Proliferation

Cellular genesis and demise are governed by tightly regulated, genetically programmed pathways [112]. In normally developing tissues, these processes remain under homeostatic control, whereas some cells undergo clonal expansion and acquire additional genetic and phenotypic alterations that enhance their survival and proliferation [113]. These transformations are primarily driven by multifactorial interplay among epigenetic and metabolic reprogramming, microenvironmental cues, and other determinants. The resultant alterations in gene expression or mutations confer competitive advantages over normal cells, leading to the clonal expansion of tumor subpopulations with acquired malignant traits. Ultimately, iterative cycles of mutation, selection, and proliferation culminate in tumor progression and oncogenic sequelae [114, 115].

Certain circRNAs modulate tumor proliferation primarily through dysregulating metabolic pathways. For instance, Mo et al. identified a novel circRNA, circRNF13, which is pathologically downregulated in NPC tissues and cell lines. Mechanistically, circRNF13 upregulates SUMO2 protein expression, suppresses glycolytic flux, and consequently inhibits the AMPK–mTOR signaling axis, ultimately attenuating tumor proliferation [116]. In HCC studies, Mei et al. demonstrated that circEPB41(2) interacts with the m^6^A demethylase FTO to modulate mRNA stability of histone deacetylase SIRT6, thereby facilitating transcriptional activation of lipid metabolism‐associated genes. Experimental validation revealed that circEPB41(2) knockdown significantly suppresses HCC cell proliferation and attenuates tumor growth in vivo [117]. Furthermore, circRNAs modulate tumor growth by regulating cell cycle progression and apoptosis. Yu et al. identified that the TP53 gene produces circp53 (hsa_circp53_0041947) with coding function. Clinical analyses revealed significant downregulation of circp53 in multiple myeloma patients, correlating with reduced survival. Mechanistically, the translation product circp53‐209aa activates mitochondrial apoptosis through a unique pathway. The researchers also engineered an EV‐based delivery platform (E7/Her2–Lamp2b–EV) for targeted circp53 delivery, which potently suppressed xenograft tumor growth and enhanced bortezomib therapeutic efficacy [102].

### 5.2 The Role of CircRNAs in Tumor Immune Escape

The immune system plays a crucial role in identifying and removing aberrant cells, preserving cellular homeostasis, and preventing malignant transformation [118]. Tumor immune evasion constitutes a pathological condition where tumors bypass immune surveillance mechanisms to sustain uncontrolled growth. This process involves multiple strategies such as altered antigen presentation, creation of immunosuppressive microenvironments, and suppression of immune cell function, collectively enabling cancer cell survival [119, 120, 121]. Emerging evidence highlights specific circRNAs as key modulators of these immune evasion pathways.

Functionally, hsa_circ_0007991 acts as a competitive ceRNA that binds to and sequesters miR‐505‐3p, leading to increased expression of the endoplasmic reticulum chaperone CANX. This molecular interaction not only promotes tumor proliferation but also diminishes CD8^+^ T cell cytotoxicity. Additional investigations confirmed the cytoplasmic stability of this circRNA, which enhances immune evasion through dual activation of the TGF‐β receptor/SMAD2 axis and PD‐L1 signaling pathways [122]. Similarly, the highly expressed circRNA hsa_circ_0136666 in gastric cancer facilitates immune evasion by modulating the hsa_circ_0136666/miR‐375/PRKDC axis. This regulatory network promotes PRKDC‐mediated phosphorylation of PD‐L1, preventing its proteasomal degradation and resulting in abnormal PD‐L1 accumulation. Miu et al. showed that LNP‐encapsulated siRNA targeting hsa_circ_0136666 effectively reduces recruitment of immunosuppressive MDSCs and Treg cells, substantially improving the efficacy of anti‐PD‐L1 therapy [123]. Moreover, hsa_circ_0020397 binds to and neutralizes miR‐138, thereby upregulating both PD‐L1 and telomerase reverse transcriptase (TERT). This mechanism enhances tumor survival while weakening T cell‐mediated cytotoxicity. Additionally, circPIAS1 inhibits STAT1 phosphorylation, suppressing ferroptosis and consequently diminishing the effectiveness of anti‐PD‐1 treatment [124]. Targeting these circRNAs presents a promising strategy for overcoming tumor immune evasion, potentially opening new avenues for clinical cancer therapy.

### 5.3 The Role of CircRNAs in Tumor Invasion and Metastasis

Although primary tumors pose a significant threat, their danger to patients is relatively limited compared with tumor invasion and metastasis, which are major causes of clinical mortality [125, 126]. Specific circRNAs play crucial roles in metastatic progression by modulating EMT, promoting angiogenesis and lymphangiogenesis, and establishing premetastatic niches.

Liver metastasis is the most common distant metastatic pattern in CRC. Research has shown that circSATB1 promotes CRC cell metastasis both in vitro and in vivo. Mechanistically, circSATB1 orchestrates RNF25‐mediated ubiquitin‐dependent degradation of FKBP8, thereby derepressing FKBP8's inhibition of the mTOR pathway, activating EMT, and enhancing tumor invasion and hepatic dissemination [103]. Additionally, circAKT3 and circAQR facilitate tumor metastasis in prostate and thyroid carcinomas, respectively, through EMT modulation in vivo [127, 128]. Beyond EMT, tumor angiogenesis is a critical prerequisite for invasion and metastasis. Pathological angiogenesis not only supplies oxygen and nutrients to tumor cells but also aids in the clearance of metabolic waste. Jiang et al. identified significant upregulation of circFNDC3B in oral SCC, which positively correlates with lymph node metastasis. Functional analyses in vitro and in vivo demonstrate that circFNDC3B promotes vascular endothelial tubulogenesis and induces lymphatic endothelial cell migration and tube formation, thereby enhancing tumor metastasis [129]. Conversely, circFAM13B suppresses lymphangiogenesis and lymph node metastasis in NPC. Tissue microarray analysis of 255 patients revealed a 35% improvement in 5‐year survival rates among those with high circFAM13B expression, and multivariate analysis confirmed its status as an independent prognostic factor [104]. Leveraging multiomics insights into circRNA regulatory networks will facilitate the development of novel therapeutics and diagnostic biomarkers targeting tumor invasion and metastasis.

### 5.4 The Role of CircRNAs in Tumor Cell Death

Cellular death is a fundamental mechanism for maintaining tissue homeostasis, and its dysregulation is intricately linked to cancer pathogenesis [104, 130]. Targeting tumor cell death represents a promising therapeutic strategy, and growing evidence indicates that circRNAs play pivotal regulatory roles in cell death pathways. Hsa_circ_0003141 is significantly upregulated in HCC tissues, where it functions as a molecular sponge for miR‐1827, derepressing UBAP2 inhibition and thus suppressing the mitochondrial apoptotic pathway [131]. CircTFRC drives ferroptosis resistance in gastric cancer by recruiting the RBP ELAVL1 to stabilize SCD1 mRNA through 3′UTR binding. This enhances monounsaturated fatty acid synthesis, suppresses lipid peroxidation, and ultimately antagonizes ferroptosis. In xenograft models, circTFRC siRNA treatment reduced tumor volume by 58% and pulmonary metastatic nodules by 72%, highlighting its therapeutic potential for gastric cancer intervention [105].

### 5.5 The Role of CircRNAs in the Tumor Microbiome

Tumors harbor a complex microbial ecosystem comprising bacteria, fungi, and viruses, collectively termed the intratumoral microbiota. This microbial community plays a crucial role in tumor progression by modulating immune regulation, inflammatory responses, and metabolic reprogramming [132]. To date, such microbial communities have been detected in at least 33 different human cancer types [133]. Notably, broad‐spectrum antibiotic treatment induces gut dysbiosis, which in turn disrupts the TME. Employing deep sequencing combined with animal models and fecal microbiota transplantation. Zhang et al. revealed that mmu_circ_0000730 suppresses mmu‐miR‐466i‐3p to upregulate the oncogenic transcription factor SOX9 in lung cancer stem cells. These findings highlight the gut microbiota's potential to regulate cancer progression and metastasis through the IL‐11‐mediated circRNA/miRNA/SOX9 signaling axis [134]. Although emerging evidence demonstrates that gut microbiota influences neuropsychiatric disorders via specific circRNAs (e.g. circ_0001239 and circHIPK2), the role of circRNAs at the tumor‐microbiota interface remains poorly understood [135]. Further mechanistic studies are urgently needed to elucidate the functional relationships between circRNAs, microbial communities, and tumor pathogenesis.

### 5.6 CircRNA and Metabolic Reprogramming

Metabolic reprogramming is a hallmark of tumor cells, characterized by alterations in glucose, amino acid, and lipid metabolism to support rapid proliferation, stress adaptation, and microenvironmental remodeling [76]. Accumulating evidence suggests that circRNAs serve as key regulators of these metabolic pathways by functioning as miRNA sponges, interacting with proteins, or encoding short peptides [136, 137, 138].

Enhanced glycolysis is a prominent feature of metabolic reprogramming in cancer cells, enabling them to meet increased energy and biosynthetic demands. Bioinformatic analyses by Wang et al. revealed that circMAT2B is significantly upregulated in HCC tissues and cell lines. Patients with high circMAT2B expression exhibited reduced overall survival. Mechanistic studies demonstrated that circMAT2B acts as a molecular sponge for miR‐338‐3p, thereby upregulating pyruvate kinase M2 (PKM2), a key glycolytic enzyme directly targeted by miR‐338‐3p. This circMAT2B/miR‐338‐3p/PKM2 axis enhances glycolysis and promotes HCC progression [106]. In lipid metabolism, Wu et al. discovered that circACC1 stabilizes and enhances the enzymatic activity of the AMPK holoenzyme by forming a ternary complex with its regulatory β‐ and γ‐subunits. This mechanism simultaneously promotes fatty acid synthesis and oxidation, providing energy and biosynthetic precursors to sustain the rapid proliferation of CRC cells. In tumor xenograft models, circACC1 knockdown suppressed tumor growth, while its overexpression enhanced tumor progression [107]. Glutamine metabolism is one of the most extensively studied aspects of amino acid reprogramming in cancer, with multiple circRNAs identified as pivotal regulators. Yao et al. demonstrated that circ_0000003 acts as a molecular sponge for miR‐330‐3p, thereby relieving its inhibition of glutaminase (GLS). This upregulates GLS expression, enhances glutamine consumption, increases α‐ketoglutarate (α‐KG) production, and elevates ATP generation, collectively driving proliferation, migration, and invasion in tongue SCC [108]. Similarly, Wang et al. reported that circ_0000808 facilitates non‐small cell lung cancer (NSCLC) progression by sequestering miR‐1827, which releases the suppression of solute carrier family 1 member 5 (SLC1A5), a key glutamine transporter. Consequently, glutamine uptake, glutamate, and α‐KG production are increased, ultimately promoting NSCLC proliferation, migration, and invasion [109]. Although numerous circRNAs play indispensable roles in metabolic reprogramming, the complexity of metabolic networks in tumor cells necessitates further fundamental research to elucidate their context‐specific mechanisms. Such investigations are essential for establishing the foundation for clinical translation and for developing novel therapeutic strategies targeting tumor metabolism.

### 5.7 CircRNA Regulates Cellular Senescence

Cellular senescence represents a fundamental biological process that persists throughout an organism's lifespan, where maintaining the delicate equilibrium between senescent cell clearance and new cell proliferation is critical for physiological homeostasis. Emerging evidence highlights this process as a pivotal pathogenic driver in diverse diseases, ranging from neurodegenerative disorders and cardiovascular conditions to carcinogenesis, with circRNAs emerging as key players within these regulatory networks [139, 140, 141].

As established in prior research, specific circRNAs exert their biological functions through protein interactions. Notably, circDNA2v demonstrates significant upregulation in colorectal cancer, where it binds to IGF2BP3 protein and protects it from ubiquitin‐mediated degradation, thereby prolonging its half‐life. The stabilized IGF2BP3 subsequently binds to and stabilizes proto‐oncogene c‐Myc mRNA, amplifying its expression. Importantly, circDNA2v inhibition results in c‐Myc downregulation and triggers tumor cell senescence [140]. Additionally, circFoxo3 interacts with crucial cell cycle regulators such as cyclin‐dependent kinase 2 (CDK2) and the CDK inhibitor p21, forming a ternary circFoxo3–p21–CDK2 complex that impedes cell cycle progression from G1 to S phase, ultimately promoting cellular senescence [142]. Certain circRNAs contribute to senescence through molecular sponge mechanisms. Liang et al. revealed that circCDYL functions as a molecular sponge for miR‐1275, consequently upregulating ATG7 and ULK1 expression to enhance autophagy and accelerate breast cancer progression [110]. However, the dual role of senescent tumor cells, exhibiting both protumorigenic and antitumorigenic effects, is mediated by complex interactions between various senescence‐associated secretory phenotype factors and the immune microenvironment. While targeting circRNAs to induce tumor cell senescence presents a promising antitumor strategy, further functional characterization of relevant circRNAs remains essential to validate their clinical potential [143].

### 5.8 CircRNA Regulates Epigenetic Modifications

Epigenetic modifications are heritable alterations in gene function that occur without changes to the underlying DNA sequence. These include DNA and RNA methylation, histone modifications, and posttranscriptional regulation mediated by ncRNAs. Such mechanisms allow cells sharing identical genetic backgrounds to acquire distinct functional identities. Increasing evidence indicates that circRNAs are integral to these processes through complex interactions with DNA, proteins, and other RNAs, thereby exerting profound effects on the initiation and progression of tumors [144].

Regarding DNA methylation regulation, FECR1, a circRNA derived from the FLI1 gene, has been shown to bind CpG islands in the promoters of both FLI1 and DNMT1. By recruiting the TET1 demethylase, FECR1 promotes site‐specific DNA demethylation and transcriptional activation. Simultaneously, FECR1 suppresses DNMT1 expression, leading to reduced global DNA methylation levels and enhanced invasiveness of breast cancer cells [145]. Additionally, certain circRNAs can modulate chromatin architecture and influence gene transcription by regulating histone modifications. Chromatin immunoprecipitation sequencing data indicate that circZKSCAN1 alters chromatin states through the repression or activation of specific histone marks, thereby contributing to the progression of melanoma [146]. m^6^A is the most prevalent internal modification in eukaryotic mRNAs, and certain circRNAs also undergo m^6^A modification. This epitranscriptomic mark significantly influences circRNA stability and function. For example, in NSCLC, m^6^A‐modified circIGF2BP3 (hsa_circ_0079587) downregulates the immune checkpoint protein PD‐L1, thereby impairing antitumor immune responses. Conversely, m^6^A‐modified circNSUN2 promotes colorectal cancer liver metastasis by facilitating the assembly of a circNSUN2/IGF2BP2/HMGA2 ternary complex [147]. Super‐enhancers (SEs), genomic regions with exceptionally strong enhancer activity, have been implicated in the regulation of circRNA biogenesis. SEs are marked by dense occupancy of transcription factors, cofactors, and histone modifications such as H3K27ac. A recent study demonstrated that SEs enhance both the diversity and abundance of circRNA isoforms from host genes by promoting RBP recruitment and modulating transcriptional elongation. Importantly, the same study identified a pan‐cancer tumor‐suppressor signature termed CircRNA Isoform Reduction from Shortened Enhancers in Cancer, which exhibits strong prognostic value in lung ADC [148].

Collectively, these findings highlight the central role of circRNAs in epigenetic and transcriptional regulation. By bridging DNA, RNA, and protein regulatory layers, circRNAs contribute to tumor initiation, progression, and metastasis, underscoring their potential as diagnostic biomarkers and therapeutic targets.

## 6 CircRNAs Serve as Promising Biomarkers for Early Tumor Diagnosis

### 6.1 Temporal Dynamics of CircRNA Expression in Early Tumor Progression

The development of cancer is a complex and prolonged process, typically progressing from normal tissue through inflammatory responses and precancerous lesions to early‐stage cancer, often spanning decades [149]. Consequently, investigating the molecular mechanisms underlying this multistep progression is critically important.

CRC currently has the highest incidence among gastrointestinal malignancies and is strongly associated with ulcerative colitis (UC) [150]. In clinical practice, early detection primarily relies on endoscopic examination; however, due to limitations in patient compliance and variability in endoscopist expertise, a significant proportion of early‐stage tumors remain undiagnosed. Recent research has revealed that circRNAs exhibit stage‐specific expression dynamics with distinct characteristics during the progression from inflammatory bowel disease to colorectal tumorigenesis. In UC patients, the downregulation of multiple circRNAs, including circCCND1, circ_0001021, and circHECTD1, is observed in colonic epithelial tissues. Mechanistic studies show that these circRNAs contribute to intestinal epithelial barrier integrity [151, 152, 153]. Additionally, circHIPK2 is moderately elevated in intestinal epithelial cells during active UC and further increases in the colonic adenoma stage. Mechanistic studies demonstrate that FUS mediates the formation of a circHIPK2–EIF4A3 complex, which promotes TAZ translation and ultimately drives epithelial cell proliferation in colitis and colorectal carcinogenesis [111]. Similarly, cervical cancer development progresses through a well‐defined multistep continuum, evolving from normal cervical epithelium to HPV infection/inflammation, cervical intraepithelial neoplasia (CIN), and ultimately early‐stage invasive carcinoma [154]. Notably, recent research has shown a stepwise upregulation of circ‐LDHA expression across normal cervical tissues, CIN lesions, and cervical carcinomas. This circRNA maintains persistent elevation under HPV infection and *Acinetobacter lwoffii* costimulation, thereby orchestrating malignant transformation via the circ‐LDHA/miR‐34a/HMGB1 signaling axis [155].

### 6.2 Noninvasive Detection of CircRNA

**FIGURE 5:**
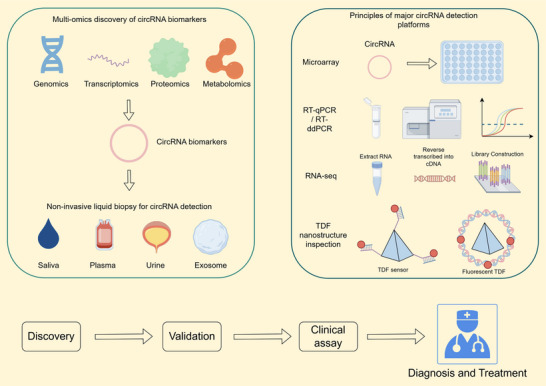
Flow chart of clinical translation research on circRNA. Multiomics approaches are employed to identify candidate circRNA biomarkers that can be detected in noninvasive liquid biopsy specimens, including saliva, plasma, urine, and exosomes. These biomarkers are subsequently quantified using various detection platforms, such as microarrays, RT‐qPCR/RT‐ddPCR, RNA‐seq, and nanostructure‐based sensors. Following validation and the development of clinical assays, these biomarkers may ultimately aid in disease diagnosis and treatment.

Advances in multiomics technologies, including genomics, proteomics, and metabolomics, have facilitated the discovery and clinical translation of numerous promising biomarkers for oncology [156]. Research indicates that circRNA expression is closely associated with tissue homeostasis, cellular dynamics, and tumorigenesis, positioning circRNAs as viable biomarkers for various pathologies, including cancer [26, 157]. In clinical practice, noninvasive biomarkers and liquid biopsy techniques are widely used for real‐time monitoring of disease progression and therapeutic response [158]. CircRNAs exhibit stable expression and relatively high abundance in biofluids such as saliva, plasma, serum, and exosomes, making them ideal candidates for noninvasive liquid biopsy‐based cancer detection [159, 160]. Several primary noninvasive methods for circRNA detection include microarray analysis, RNA‐sequencing (RNA‐seq), reverse transcription quantitative polymerase chain reaction (RT‐qPCR), and reverse transcription droplet digital PCR (RT‐ddPCR) (Figure 5).

Microarray‐based circRNA detection relies on specific hybridization between predesigned oligonucleotide probes and target circRNAs. The process begins with the selective elimination of linear RNAs via RNase R digestion, followed by hybridization of fluorescence‐labeled circRNAs to immobilized BSJ‐targeting probes on the microarray chip [161]. Scanning the fluorescence signal intensity enables semi‐quantitative analysis of circRNA expression profiles [162]. While microarrays are cost‐effective and efficient, their ability to detect new transcripts is limited due to predesigned templates, and their accuracy is compromised at extremely low or high gene expression levels. Consequently, microarrays are often used as a preliminary screening step before conducting quantitative circRNA analyses.

RNA‐seq has become a prevalent tool in molecular biology research. Unlike microarrays, RNA‐seq can discover novel circRNAs and facilitate large‐scale testing in clinical samples. The core principle involves identifying unique back‐splicing patterns derived from the circular conformation of circRNAs. This process starts with digesting total RNA with RNase R to selectively degrade linear RNAs, followed by purification and construction of strand‐specific whole‐transcriptome libraries. Subsequent fragmentation of RNA templates enables the synthesis of double‐stranded cDNA fragments for sequencing. Advanced algorithms, such as CIRI and CIRI‐deep, have been developed for circRNA detection. CIRI uses a BWA–MEM‐based circular alignment methodology to identify BSJs by detecting soft‐clipping signals in paired‐end mapping data. Its latest version incorporates a convolutional neural network (CNN) module to filter false positives through sequence‐context features, such as splice site conservation and RNA secondary structure [163]. However, due to the absence of poly(A) tails in circRNAs, conventional transcriptomic library preparation methods such as polyA enrichment fail to efficiently capture them, resulting in the loss of circRNA expression information in most traditional single‐cell and spatial transcriptomic datasets. To address this limitation, researchers have developed CIRI‐deep, a deep learning model that circumvents dependence on traditional back‐splicing signals and instead utilizes specific cis‐regulatory elements and trans‐acting factors as input features. Evaluation results demonstrate that CIRI‐deep enables reliable prediction of differentially spliced circRNAs in transcriptomic sequencing data, thereby achieving accurate cell‐type‐specific circRNA resolution at single‐cell and spatial levels [164].

The NanoString nCounter platform enables direct digital counting of RNA molecules without amplification, providing highly specific and sensitive quantification. By employing a pair of probes (a capture probe and a reporter probe) designed to span the BSJ, the technology ensures precise discrimination between circRNAs and their linear counterparts. During solution hybridization, these probes bind specifically to the target circRNA, forming a stable hybrid complex. After purification and immobilization, the complexes are immobilized onto a cartridge for subsequent digital imaging and quantification [165]. This amplification‐free approach avoids PCR‐induced biases, delivering more direct and reliable measurements with high specificity and reproducibility. Furthermore, the platform supports multiplex detection, enabling simultaneous quantification of hundreds of circRNAs in a single run. Its compatibility with diverse clinical specimens, including plasma, urine, and formalin‐fixed paraffin‐embedded tissues, underscores its translational potential [166]. Despite these advantages, the NanoString nCounter system is relatively costly, especially for large sample cohorts, and relies on custom‐designed probes specific to the BSJ, which require extended design and synthesis timelines. These factors hinder its widespread adoption, particularly in resource‐limited laboratories [167].

RT‐qPCR is extensively used for the detection and validation of circRNAs. It employs different primers flanking the BSJ locus to amplify circRNA fragments and detect circRNAs. A standard procedure involves treating total RNA extracted from the sample with RNase R, followed by RT to generate the complementary DNA of the circRNA, and subsequently determining and analyzing the fluorescent signal [168]. This technology offers superior sensitivity, detection speed, and time efficiency for circRNA detection, making it well suited for clinical laboratories. However, RT‐qPCR has significant errors in accurately quantifying trace nucleotides in plasma and may overestimate circRNA concentrations due to strand displacement and roll‐over replication during RT [169, 170]. To address these challenges, an assay utilizing RT‐ddPCR was developed. RT‐ddPCR is an emerging assay technology with higher sensitivity and accuracy than conventional PCR, which has been demonstrated to be the most suitable method for quantifying circRNA in tissues and plasma [168]. RT‐ddPCR is unaffected by standard curves and amplification kinetics, allowing for absolute quantification and avoiding the impact of differences in PCR amplification efficiency. It requires a small sample size and exhibits high detection sensitivity, making it appropriate for clinical samples [171]. However, these conventional methods necessitate skilled personnel, specialized laboratory facilities, and processing times exceeding 24 h, making rapid and sensitive circRNA detection challenging. To address this, Yao et al. developed a strategy using tetrahedral DNA frameworks for selective recognition and high‐affinity capture of intact circRNAs. This approach involves constructing multivalent DNA nanostructure probes spatially complementary to circRNA conformations. By conjugating two‐photon fluorophores to the probes, it enables real‐time imaging of freely migrating circRNAs in living systems while simultaneously facilitating dynamic tracking during circRNA delivery processes [172].

Exploiting the unique circular structure of circRNAs, researchers have developed a method that selectively amplifies circular templates without amplifying linear RNAs, known as RT‐rolling circle amplification (RT‐RCA). In this approach, circRNAs are first reverse‐transcribed into cDNA using specifically designed primers, followed by circularization of the cDNA template via ligases or similar enzymes. DNA polymerase then continuously extends along the circular template, generating long tandem repeat sequences. The amplified products are subsequently detected [173]. Zhu et al. combined RT‐RCA with a carefully designed networked hybridization chain reaction (HCR) to develop a circRNA detection method based on isothermal networked HCR. In this approach, the networked structure forms a stable mesh with RT‐RCA products containing multiple tandem repeats under isothermal conditions, significantly enhancing the fluorescent signal. Experimental results demonstrated that the optimized networked HCR system exhibits higher detection efficiency and excellent selectivity for DNA strands containing multiple repeat sequences. Notably, this method allows accurate detection of specific circRNAs in authentic biological samples without RNase R enrichment, providing a simple and effective platform for the detection of low‐abundance circRNAs [174]. However, this detection method has so far been limited to circRNAs in specific tissues, and the impact of abundant interfering substances in complex biological samples, such as blood or saliva, remains unclear. Future studies should aim to expand the application of RT‐RCA to various biological specimens, including body fluids and feces, while enhancing its resistance to interference. Additionally, improving the efficiency of simultaneous detection of multiple circRNAs will be essential to meet the demands of high‐throughput and complex sample analyses [173, 175].

## 7 CircRNA and Encoded Proteins as Therapeutic Targets

There is increasing evidence that circRNAs and the proteins they encode function as ceRNAs by sequestering miRNAs and upregulating downstream genes. This mechanism ultimately contributes to various aspects of tumor progression, metastasis, and enhances therapeutic resistance by inhibiting cell death processes such as apoptosis and ferroptosis. Consequently, these specific circRNAs and their associated pathways are anticipated to serve as novel therapeutic targets, offering additional opportunities for clinical intervention (Table 2).

**TABLE 2:** The carcinogenic mechanism of CircRNA in various types of tumors and cross‐model validation.

Cancer type	Experimental model	Target circRNA	Specific pathway	References
Gastric cancer	Cell line, CDX	circMTHFD2L	circMTHFD2L/CM‐248aa/SET/PP2A/AKT;ERK;P65	[176]
Gastric cancer	Cell line, CDX	circHIPK3	circHIPK3/miR‐508‐3p/Bcl‐2/beclin1/SLC7A11	[177]
Nasopharyngeal carcinoma	Cell line, CDX	circFIP1L1	circFIP1L1/miR‐1253/EIF4A3/PTEN	[178]
Lung adenocarcinoma	Cell line, CDX	cEMSY	cEMSY/TDP‐43/mtDNA/cGAS/STING	[179]
Breast cancer (triple negative)	Cell line, PDO	circPVT1	circPVT1/miR‐33a‐5p/c‐MYC/GLS1	[180]
Ovarian cancer	Cell line, CDX, PDO	hsa_circ_0010467	AUF1/hsa_circ_0010467/miR‐637/LIF/STAT3	[181]
Breast cancer (HER2‐low)	Cell line, PDX	crVDAC3	crVDAC3/HSPB1	[182]
Hepatocellular carcinoma	Cell line, CDX, PDX	circMDK	circMDK/miR‐346;miR‐874‐3p/ATG16L1/PI3K–AKT–mTOR	[183]
Breast cancer (HER2‐negative)	Phase I clinical trial	CircFAM53B	circFAM53B/IRES‐driven translation/FAM53B‐219aa peptide/HLA‐I&II presentation/antigen‐specific T cell activation/tumor killing	[184]
Large‐cell lymphoma (ALK‐positive anaplastic)	Clinical trial	/	/	[185]

### 7.1 Therapeutic Targeting Across Models

Numerous studies utilizing in vitro cell cultures and cell line‐derived xenograft (CDX) models have established that targeting circRNAs or their encoded proteins can effectively suppress tumor progression and metastasis, while also reversing tumor cell resistance to chemotherapy, radiotherapy, and immunotherapy. For instance, CM‐248aa, a protein encoded by circMTHFD2L, has been shown to inhibit gastric cancer cell proliferation and metastasis when overexpressed in gastric cancer cell lines, CDX models, and lung metastasis models [176]. Research on cisplatin resistance mechanisms revealed that silencing circHIPK3 in cisplatin‐resistant gastric cancer cells and CDX models suppresses tumor progression and metastasis by activating the miR‐508‐3p/Bcl‐2/beclin1/SLC7A11 axis to promote ferroptosis, thereby reducing cisplatin resistance [177]. Similarly, elevated circFIP1L1 expression stabilizes PTEN mRNA via the miR‐1253/EIF4A3 axis, inducing apoptosis in NPC cells and enhancing radiosensitivity, as demonstrated in CDX models [178]. Notably, the circRNA cEMSY has been identified as an immunogenicity enhancer both in vitro and in vivo (in immunosuppressed tumor models), where it triggers immunogenic cell death in lung ADC cells, thereby amplifying antitumor immune responses and potentiating the efficacy of immune checkpoint inhibitors [179].

Preclinical investigations employing patient‐derived organoid (PDO) and patient‐derived xenograft (PDX) models have further underscored the clinical translational potential of targeting circRNA‐related pathways in cancer therapy, although no related drugs have advanced to clinical trials. For example, circPVT1 depletion sensitizes breast cancer cells to glutaminase inhibitor‐induced cytotoxicity through the miR‐33a‐5p/Myc/GLS1 axis, a finding validated in TNBC‐derived organoids [180]. In ovarian cancer cells, CDX models, and PDOs, silencing hsa_circ_0010467 markedly enhances cisplatin's antitumor effects by modulating the AUF1/hsa_circ_0010467/miR‐637/LIF/STAT3 axis [181]. Additionally, in HER2‐low PDX models, paritaprevir binds VDAC3‐derived circRNA, promoting HSPB1 protein ubiquitination and degradation while elevating ferroptosis levels, thereby overcoming trastuzumab deruxtecan resistance in HER2‐low breast cancers [182].

Through a comprehensive approach involving four HCC animal models (subcutaneous, metastatic, PDX, and orthotopic), Du et al. demonstrated that the poly(β‐amino ester)‐based circMDK siRNA delivery system (PAE–siRNA complex) effectively silences circMDK, inhibiting HCC progression and metastasis in vivo [183].

### 7.2 CircRNA Reshaping CAR‐T Therapeutic Areas

CircRNAs are reshaping landscape and practicing CAR‐T therapy at multiple levels. Numerous studies have shown that, at the adaptive immunity level, circRNAs can impair T‐cell function by upregulating immune checkpoint molecules or diminish their antitumor activity by reducing T‐cell infiltration into tumor tissues [147, 186, 187, 188]. At the innate immunity level, circRNAs can induce macrophage polarization toward the M2 (tumor‐promoting) phenotype and suppress the tumor‐killing function of NK cells [78, 189]. Given that CAR technology functions by activating these immune cells, circRNAs may attenuate CAR‐T cell and associated immune cell activity through these mechanisms, thereby diminishing the antitumor efficacy of CAR technology [63].

Conversely, a growing body of research highlights the unique application potential of circRNAs in adoptive cell therapy. On one hand, circRNAs can directly participate in CAR‐T preparation processes. Their covalently closed‐loop structure confers high stability and precise gene regulatory functions, enhancing the persistence and specificity of CAR‐T cells in cancer treatment. Specifically, compared with traditional mRNA‐based CAR‐T preparation methods, using circRNAs as a delivery system for precise insertion of CAR transgenes offers higher gene integration efficiency, enhanced gene tagging accuracy, and reduced production costs [190, 191, 192]. Additionally, circRNAs can minimize off‐target effects by reducing nonspecific binding of CAR‐T cells to healthy tissues through acting as miRNA sponges, or by enhancing the targeting accuracy of CAR‐T cells via increasing the expression of tumor‐specific receptors on CAR‐T cells and upregulating the levels of target antigens (such as HER2, CD19) on tumor cell surfaces [190, 193, 194]. When delivered in a vaccine, circRNAs can enhance the therapeutic efficacy of CAR‐T cells by regulating CAR‐T cell proliferation, infiltration, and exhaustion processes. Furthermore, they can optimize CAR‐T therapy by providing supplemental antigens or functioning as adjuvant enhancers.

A recent study demonstrated that delivering circRNACAR encoding anti‐HER2 CAR alongside immunocellular‐targeting lipid nanoparticles (LNPs) promotes the in vivo formation of a panCAR system encompassing CAR‐T, CAR‐NK, and CAR‐macrophage cells, significantly inhibiting tumor growth. The combined use of circRNA vaccines encoding the corresponding HER2 antigen enhances circRNACAR‐mediated antitumor therapy by increasing CAR‐T cell proliferation, promoting CAR‐T cell infiltration, and attenuating CAR‐T cell exhaustion [195]. Numerous prior studies have confirmed that circRNAs can reduce T‐cell exhaustion by acting as miRNA sponges, interacting with RBPs, regulating activation‐induced cell death, and activating T‐cell survival‐related transcription factors [194, 196, 197]. They also create a favorable environment for CAR‐T cell infiltration and activation by downregulating extracellular matrix protein expression and modulating cytokine and chemokine levels within the TME [194]. Moreover, circRNA vaccines may provide additional antigenic signals by encoding tumor‐specific antigens, thereby enhancing endogenous T‐cell immunity to assist CAR‐T cells in eliminating antigen‐deficient tumor cells [69, 198, 199]. CircRNAs may also function as “adjuvant enhancers”; for example, circFOREIGN activates type 1 conventional dendritic cells (cDC1s), enhancing antigen presentation efficiency, which may boost CAR‐T cell activation and proliferation capacity [198, 200]. Precisely due to circRNA's stable expression patterns and multifaceted regulatory functions, it is highly suitable as a biomarker for predicting CAR‐T outcomes [201].

### 7.3 Delivery Systems

Currently, cancer treatments utilizing circRNAs can be categorized into three primary approaches. The first approach involves delivering siRNAs, antisense oligonucleotides, or CRISPR–Cas13a to silence oncogenic circRNAs. The second approach entails constructing and delivering overexpression vectors for tumor‐suppressing circRNAs. The third approach involves delivering circRNAs that encode functional proteins to participate in cancer therapy [159].

The key to these circRNA‐based cancer therapies lies in the precise and efficient delivery of the target molecules to the desired tissues or cells. Common delivery systems for transporting circRNAs include LNPs, exosomes, viruses, peptide vectors, and composite nanoparticles [202]. Most studies have utilized adenoviral and lentiviral vectors as viral delivery systems to target circRNAs; however, due to their limited gene‐carrying capacity, high costs, and potential biosafety concerns, an increasing number of studies are now focusing on LNPs or engineered exosomes for circRNA delivery [203].

#### 7.3.1 CircRNA Vaccine

CircRNA vaccines represent a novel class of vaccines capable of inducing the expression of antigens that elicit adaptive immune responses. The circRNAs used in these vaccines primarily possess protein‐coding functions. Compared with traditional mRNA vaccines, circRNA vaccines typically produce higher and more sustained antigen levels for several reasons.

First, circRNAs are covalently closed loop structures that resist degradation by RNA exonucleases, making circRNA vaccines structurally stable and easier to store [199]. Second, the lack of free ends in circRNAs avoids recognition by innate immune receptors such as RIG‐I, thereby reducing immunogenicity and cytotoxicity [204, 205]. Third, the roll‐over translation mechanism of circRNAs prolongs the expression time, making them an ideal vector for efficient protein expression [204, 205].

#### 7.3.2 LNP Delivery System

LNPs have emerged as a predominant delivery vehicle for circRNA‐targeting therapeutics. These nanoparticles typically comprise four key components: ionizable cationic lipids, phospholipids, cholesterol, and polyethylene glycolated lipids [206]. The ionizable cationic lipids facilitate RNA encapsulation within LNPs through electrostatic interactions with negatively charged RNA molecules [207]. In a notable study, Xu et al. employed a high‐throughput combinatorial approach to develop optimized LNPs for efficient circRNA delivery to lung tumors. Their findings demonstrated that a single intratumoral injection of IL‐12 circRNA‐loaded LNPs significantly enhanced immune cell infiltration within the TME and induced marked tumor regression in the LLC1 murine tumor model [208]. Furthermore, Zhou et al. pioneered an innovative therapeutic platform utilizing in vitro circRNA (ivcRNA) for osteoarthritis treatment. Their strategy involves local intra‐articular administration of LNP‐encapsulated ivcRNA encoding therapeutic proteins. This delivery system exhibits exceptional stability and translational efficiency, enabling sustained expression of therapeutic proteins such as MSI2 and SOX5—outperforming m^1^Ψ‐modified linear mRNA in both durability and protein yield. The approach shows significant efficacy in mitigating osteoarthritis progression, representing a promising advancement in circRNA‐based therapeutic strategies [209].

#### 7.3.3 Exosome Delivery System

Exosomes are endogenous EVs actively secreted by cells. Tumor‐derived exosomal circRNAs have been demonstrated to regulate immune cell antitumor activity and modulate immune checkpoint protein expression, while also promoting angiogenesis, participating in cancer metabolism, and contributing to tumor proliferation, metastasis, and drug resistance. Notably, various cellular components within the TME—including immune cells, CAFs, adipocytes, and mesenchymal stem cells—can influence tumor progression through exosomal circRNA secretion. These properties make exosomal circRNAs promising diagnostic and prognostic biomarkers for diverse tumor types [159].

Beyond naturally occurring exosomal circRNAs, engineered exosomes have emerged as effective vehicles for delivering therapeutic agents that directly target circRNA functions. Compared with synthetic delivery systems such as LNPs and nanoparticles, exosomes exhibit superior biocompatibility, reduced immunogenicity, and enhanced chemical stability, rendering them highly suitable as delivery vectors [210, 211]. For instance, Wang et al. developed exosomes loaded with si‐ciRS‐122, which effectively suppressed colon cancer cell proliferation and reversed oxaliplatin resistance [212].

### 7.4 Synthesis of CircRNA in Therapy

The large‐scale synthesis of circRNA is crucial for advancing the widespread adoption of circRNA therapeutics.

In vitro circRNA synthesis primarily employs three methods: chemical synthesis, enzyme‐mediated synthesis using DNA or RNA ligases, and nuclease‐based approaches relying on self‐splicing introns [213]. Chemical synthesis is suitable for producing small circRNAs of high purity but involves toxic reagents, poses potential biosafety risks, and is characterized by low efficiency and high cost. Enzymatic synthesis using DNA or RNA ligases is more appropriate for preparing medium‐to‐long circRNAs, offering advantages such as high cyclisation efficiency, minimal introduction of extraneous sequences, and low immunogenicity. A recent study developed an in vitro circularization strategy based on cis‐acting ligase ribozyme (RzL). RzL autonomously catalyses RNA circularization during transcription without GTP involvement and without by‐product generation, significantly enhancing circRNA synthesis efficiency and product purity [214]. Nuclease‐based approaches using self‐splicing introns are suitable for synthesizing circRNAs of varying sizes (up to 5 kb). Their reaction conditions are straightforward, making them suitable for large‐scale production, and the synthesized circRNA products are highly safe. Wang et al. constructed a novel chimeric permuted intron‐exon system. By engineering type I self‐splicing introns, they significantly enhanced the circularization efficiency of in vitro circRNA synthesis [215]. Du et al. developed a novel circRNA synthesis method based on trans‐Rzl‐based circularization, enabling efficient preparation of long‐chain circRNAs exceeding 8000 nt. This approach not only eliminates the need for bacterial sequence introduction but also supports the simultaneous incorporation of multiple RNA modifications. This system employs virus‐derived IRES for rolling‐circle translation (RCT), enhancing protein expression efficiency by over 7000‐fold [216]. Researchers have also proposed CIRC (complete self‐splicing intron for RNA circularization), a novel intron‐mediated circRNA synthesis method. Utilizing complete type I and type II introns, it achieves efficient, rapid RNA circularization while producing scarless, low‐immunogenicity circRNAs [217]. Furthermore, Zhu et al. designed a novel circRNA‐promoting RNA (cpRNA) that targets the flanking reverse complementary matches (RCMs) within pre‐mRNAs to enhance exon circularization and thereby promote circRNA biogenesis. Additionally, cpRNA specifically upregulates tumor‐suppressive circRNAs such as circZKSCAN1 and circSMARCA5, leading to the inhibition of HCC cell proliferation and migration, and offering a potential therapeutic strategy for diseases associated with low circRNA expression. However, its effectiveness is limited to circRNAs containing RCMs, which restricts its broader applicability [218].

Beyond in vitro synthesis, circRNA can also be generated in vivo. For instance, the nuclease‐based Tornado system efficiently produces intracellular circRNA, and when combined with virus‐like particles (VLPs), it enables sustained protein expression [219]. Further studies report that the Tornado system (twister‐optimized RNA for durable overexpression) enables efficient circRNA synthesis within mammalian cells, exhibiting significantly higher efficiency than conventional backsplicing methods. Using VLPs delivery systems, it achieves more sustained target protein expression, offering novel strategies for circRNA in vivo applications [189]. Tong et al. developed two novel circRNA synthesis strategies: type II intron‐mediated in vitro circularization and in vivo circularization utilizing the intracellular RtcB ligase. They established an efficient purification method without requiring high‐performance liquid chromatography, significantly enhancing circRNA yield and purity while reducing immunogenicity [220].

CircRNA structures typically comprise IRES, coding regions, and regulatory hairpin loops. Optimizing these structures enhances protein expression, reduces immunogenicity, and improves stability, ultimately boosting the overall efficacy of circRNA therapeutics [213]. For example, Chen et al. achieved a several‐hundred‐fold increase in circRNA protein yield by systematically optimizing five key components of synthetic circRNAs: the vector topology, 5′ and 3′ UTRs, IRES, and synthetic aptamers recruiting translation initiation machinery. Embedding an eIF4G‐recruiting aptamer (Apt‐eIF4G) within a specific IRES domain was found to significantly enhance circRNA translation efficiency [221].

The purification process for synthetic circRNA is critical for its clinical applications. A team recently developed a multistep purification strategy combining enzymatic treatment (RNase R) with cellulose chromatography (e.g., WMC), which efficiently removes immunogenic impurities (such as dsRNA and 5'‐ATP) from synthetic circRNA. This significantly reduces innate immune responses and increases circRNA yield by over tenfold. CircRNA purified via this strategy demonstrated over 300% efficiency in induced pluripotent stem cell reprogramming and achieved sustained, potent antitumor expression in CAR‐T cells, establishing a high‐quality RNA platform for regenerative medicine and cancer immunotherapy [222].

Currently, several clinical trials are exploring the therapeutic potential of specific circRNAs in cancer treatment. A clinical trial (NCT06530082) is currently evaluating the safety and tolerability of the CircFAM53B‐219aa DC vaccine, both as a monotherapy and in combination with camrelizumab, for the treatment of HER2‐negative advanced breast cancer. Previously, the same research team demonstrated the tumor‐suppressive effects of CircFAM53B‐219aa in vitro and in animal models [184]. Furthermore, an ongoing clinical trial (NCT05934045) is investigating the role of circRNAs as circulating biomarkers for predicting treatment resistance in ALK‐positive anaplastic large‐cell lymphoma, as well as their potential utility as therapeutic targets [185].

## 8 Opportunities in the Clinical Translation of CircRNAs

### 8.1 Applications and Prospects of CircRNA‐Based Vaccines

Currently available vaccines fall into five main categories: inactivated vaccines, viral vector vaccines, DNA vaccines, mRNA vaccines, and emerging circRNA‐based vaccines, each representing distinct technological platforms for immunization strategies. Traditional inactivated vaccines, exemplified by inactivated hepatitis B vaccines, are produced using physical and chemical methods to neutralize pathogens. These vaccines offer high safety profiles, enhanced stability, and straightforward manufacturing processes, though they exhibit relatively weaker immunogenicity [223, 224]. Viral vector vaccines, which use replication‐deficient adenoviral vectors to deliver pathogen antigen genes, provide advantages such as convenient storage conditions and efficient delivery. However, pre‐existing immunity can compromise their clinical efficacy [225]. DNA vaccines benefit from intrinsic DNA stability and the elimination of ultra‐low temperature storage requirements, but the potential for genomic integration poses risks for germline transmission [226]. Due to these limitations, the first three vaccine platforms have constrained applicability, positioning RNA‐based therapeutics at the forefront of both fundamental research and clinical translation.

mRNA vaccines, delivered via LNPs encapsulating antigen‐encoding transcripts, undergo cytoplasmic translation to elicit potent immunostimulatory effects, including robust antigen‐specific humoral and cellular immune responses that confer broad and durable immunity [227]. Critically, their rapid development cycle, requiring only genetic sequence information, enables prompt deployment during pandemics such as COVID‐19. However, mRNA vaccines also carry potential risks, as demonstrated by a study showing that the in vitro incorporation of N1‐methylpseudouridine (m1Ψ) into mRNA can induce ribosomal frameshifting, leading to the production of frameshifted products during the translation of BNT162b2 vaccine mRNA in both murine models and human recipients [228].

The covalently closed circular architecture of circRNAs confers high stability with extended half‐lives compared with linear RNAs. During translation, circRNAs utilize IRES to initiate RCT, enabling sustained generation of repetitive antigen epitopes that enhance immunogenicity while reducing dsRNA production. Consequently, circRNA‐based vaccines represent a rapidly advancing research domain [199].

In cancer immunotherapy, Lin et al. developed the world's first intranasal circRNA vaccine by encapsulating circRNAs in LNPs to elicit localized mucosal immunity. This vaccine induced potent antitumor T cell responses in animal models, specifically eliminating pulmonary tumors in mice while mitigating systemic adverse effects typically associated with intravenously administered mRNA vaccines. It also augmented CAR‐T cell responses to enhance therapeutic efficacy against tumor cells expressing specific tumor‐associated antigens [198]. Furthermore, wang et al. pioneered a circRNA‐based neoantigen vaccine expressing the HCC‐specific neoantigen Ptpn2_I383T. This vaccine promotes DC maturation and enhances DC‐mediated T cell priming, thereby potentiating T cell cytotoxicity against tumor cells. In murine tumor models, the vaccine demonstrated robust therapeutic and prophylactic efficacy while conferring long‐term protective immunity. Synergistic strategies integrating circRNA vaccines with chemotherapeutic agents are also emerging. Zhang et al. developed a circRNA–DC vaccine encoding tumor antigens FAPα and survivin, which elicited potent CD8^+^ T cell responses and significantly suppressed tumor growth. Combining this DC vaccine with gemcitabine substantially prolonged median survival in pancreatic cancer murine models [229].

More recently, a research team from China developed a carrier‐free, scalable, and programmable circRNA platform embedded with aptamers (Apt‐circRNA). Unlike traditional delivery systems, Apt‐circRNA does not require LNPs or exogenous adjuvants; instead, it achieves targeted delivery through its built‐in aptamer structure. When administered subcutaneously as a tumor vaccine, Apt‐circRNA can efficiently target DCs and be transported to draining lymph nodes, thereby eliciting a potent and safe antitumor immune response. Preclinical and clinical studies have already demonstrated its remarkable targeted immunogenicity and excellent safety profile, establishing a solid foundation for the future development and clinical translation of aptamer‐based circRNA vaccines [230].

### 8.2 Technological Innovations: Single‐Cell CircRNA Sequencing Reveals Tumor Heterogeneity

Accurately deciphering biological systems and their dysregulation in disease requires multiparametric analysis at the single‐cell level. While single‐cell sequencing has become essential in oncology research due to advancements in multiomics profiling platforms, analyzing circRNAs at single‐cell resolution remains challenging. The key limitations include: (1) the inherently low abundance of circRNAs, which often leads to frequent dropout events in conventional single‐cell workflows; and (2) the high sequence homology between circRNAs and their linear parental transcripts, necessitating both specific enrichment strategies and sophisticated computational discrimination algorithms. Zhao et al. achieved high‐resolution profiling and comprehensive characterization of circRNAs at single‐cell resolution by systematically integrating 171 published single‐cell full‐length transcriptome datasets. Their analysis of single‐cell datasets from 20 breast cancer patients revealed significantly higher circRNA expression in both normal and malignant cells from low‐grade tumors with favorable prognosis compared with high‐grade TNBCs. CircRNA abundance progressively increased during the epithelial‐to‐intermediate EMT, but paradoxically decreased in late EMT stages. Notably, circRNA expression in cancer cells dropped below the baseline levels observed in the mesenchymal phase of normal cells, highlighting tumor‐specific circRNA heterogeneity during mesenchymal transformation [231].

### 8.3 Translational Priorities: Integrating Databases Across Cancer Types

The rapid advancement of high‐throughput sequencing technologies has facilitated the development of multiple comprehensive circRNA databases dedicated to cancer research, significantly advancing pan‐cancer circRNA investigations. By integrating circRNA expression profiles, molecular signatures, and clinical information across diverse cancer types, these databases provide invaluable resources for systematically exploring the functional roles of circRNAs in oncogenesis. Here, we summarize key publicly available circRNA databases and highlight their core features.

The CSCD database series represents one of the earliest resources dedicated to tumor‐specific circRNAs. Its upgraded version, CSCD2, compiles over 100,000 circRNAs identified from more than 1000 cancer and normal samples. Its core strength lies in constructing interactive regulatory networks that enable intuitive exploration of circRNA–miRNA/RBP interactions. Additionally, CSCD2 provides precise predictions of full‐length circRNA sequences and ORFs [232].

TCCIA is a pioneering database that bridges circRNAs with cancer immunotherapy, integrating over 4000 tumor samples from 25 independent immunotherapy cohorts. The platform employs standardized reprocessing of raw RNA‐seq data and clinical phenotypes, coupled with systematic circRNA detection, enabling users to interactively screen circRNAs significantly associated with immunotherapy response and investigate their correlations with T‐cell infiltration, immune exclusion, and other TME phenotypes [233].

MiOncoCirc is a pioneering database that systematically links circRNAs with clinical phenotypes across cancer types, integrating data from over 2000 clinical specimens, including primary tumors, metastatic lesions, and rare subtypes. Its distinctive advantage lies in directly capturing patient‐derived circRNA expression profiles within TMEs, thereby circumventing potential biases inherent in cell line models. The implementation of exon‐capture sequencing technology by the research team demonstrates enhanced circRNA detection capacity compared with conventional workflows, offering superior sensitivity and specificity to facilitate circRNA biomarker discovery [234].

Protein‐coding circRNAs play pivotal roles in disease pathogenesis, and circDB represents the first dedicated database cataloging these functional circRNAs. It integrates over 35,000 human exon‐derived circRNAs with comprehensive annotations, including exon composition, genomic origins, molecular size, sequence length, and full‐length circular transcripts. To assess protein‐coding potential, the database systematically predicts IRES elements and ORFs exceeding 300 bp, providing the two top‐ranking IRES motifs for each entry based on computational scoring algorithms [235].

Beyond these established resources, novel circRNA databases continue to emerge, including circRNADisease, which encompasses nononcological disorders, and exoRBASE, dedicated to circRNAs in human blood exosomes. These databases enable researchers to explore circRNA–disease associations while also serving as cross‐validation platforms for assessing circRNA coding potential [236].

### 8.4 The Role of Artificial Intelligence in CircRNAs

Over the past decade, artificial intelligence has ushered in a new era in medicine, becoming an indispensable tool for clinical decision support and biomedical predictive analytics. By leveraging robust computational capabilities, AI efficiently processes and analyzes the high‐throughput sequencing data generated by modern biology [237]. Various deep learning architectures enable the prediction of novel drug targets and disease risks, while AI‐driven frameworks play crucial roles in circRNA screening, sequence annotation, and functional characterization [238, 239].

#### 8.4.1 CircRNA Profiling: High‐Fidelity Identification and Association Mapping

Traditional circRNA screening methods typically require months to years from discovery to functional validation, often introducing potential biases due to technical variations [240, 241]. In contrast, AI‐driven approaches leverage deep learning frameworks to establish end‐to‐end predictive pipelines, significantly enhancing screening efficiency. This methodology begins with the curation of experimentally validated circRNA–disease associations from databases, followed by feature extraction and selection. The subsequent application of CNNs and autoencoders enables the prediction of novel circRNA–disease relationships, which are ultimately validated through targeted experiments or multiomics sequencing [242, 243].

Li et al. developed CDASOR, a deep learning framework that integrates convolutional and recurrent neural networks for circRNA–disease association prediction. CDASOR uses continuous k‐mer bag‐of‐words encoding to generate low‐dimensional circRNA sequence embeddings. These representations are processed by a 1D CNN for local feature extraction and then analyzed using bidirectional long short‐term memory (BiLSTM) networks to capture long‐range dependencies. Validation against published literature confirmed six out of ten top‐ranked predictions in case studies, demonstrating CDASOR's ability to generate biologically relevant associations [244]. However, existing deep learning models face limitations such as insufficient feature representation and high dependency on training data. To address these issues, a research team from China developed AGDFCDA, a computational framework that implements dual‐feature extraction strategies for circRNA–disease association prediction. This approach uses fully connected neural networks to reduce feature redundancy and initially extract latent representations of circRNAs and diseases. It then employs adaptive graph convolutional networks to learn comprehensive circRNA–disease embeddings for advanced feature extraction, and finally performs association prediction through multilayer perceptron architectures [245].

#### 8.4.2 Sequence Prediction: Optimizing Cyclization Efficiency and Translation Capability

Optimization of conventional linear mRNAs typically focuses on the 5′/3′ UTRs and ORFs to achieve sustained and efficient translation. In contrast, circRNAs contain IRES motifs, enabling cap‐independent translation initiation beyond ORF elements. Properly folded functional IRES motifs facilitate the recruitment of the preinitiation complex, which is essential for efficient circRNA translation [216]. Additionally, optimizing the secondary structures and codon usage in circRNA sequence design significantly enhances transcript stability while concurrently improving translational efficiency and antibody responses [246]. To address the challenges of circRNA sequence optimization, Zhang et al. developed circDesign, an algorithmic platform for circRNA structural prediction and sequence design. This platform extends their previously established AI framework, originally designed for linear mRNA sequence engineering. The model initially generates diverse mRNA sequence libraries from target amino acid sequences, then filters the ORF regions of circRNAs based on minimal free energy and codon adaptation index criteria. It assembles optimized sequences into complete circRNA molecules, evaluates IRES positional entropy and structural ensemble diversity within these constructs, and ultimately validates the designed circRNAs through in vitro and in vivo screening platforms. Experimental validation demonstrated that algorithm‐designed circRNAs exhibited significantly extended half‐lives, indicating enhanced nuclease resistance. Moreover, the CR3 circRNA isoform showed the highest abundance in both transcriptomic and ribosome footprint profiling, highlighting its efficient translation capability [247].

#### 8.4.3 Functional Prediction: Analysis of Interaction Networks

Current research on circRNA functional mechanisms primarily focuses on their interactions with proteins, making the prediction of circRNA–protein interaction sites and the assessment of their binding affinity increasingly crucial for advancing this field. Traditional experimental methods, such as CLIP‐seq, are limited by their ability to capture only single RBP binding sites within specific cellular contexts, often requiring lengthy and costly procedures [248]. Additionally, protein–RNA interactions dynamically change in response to alterations in the cellular microenvironment, while studies on protein‐mediated RNA regulation require binding information under consistent conditions [249].

Deep learning‐based prediction of circRNA–protein binding sites provides a viable alternative to overcome these economic and temporal constraints. CircRIP, a framework that integrates stacked codon‐based encoding and hybrid deep learning architectures, uses RNA sequences alone to predict RBP binding sites on circRNAs [250]. CSCRSites, another deep learning model, incorporates nucleotide positional information for the classification of cancer‐specific circRNA–RBP binding sites [251]. HCRNet, a novel end‐to‐end framework utilizing bidirectional encoder representations from transformers to contextualize circRNA sequences, demonstrates superior performance [252]. The recently proposed CRIECNN model, which combines a CNN, a BiLSTM layer, and a self‐attention mechanism for feature refinement, achieves a mean AUC of 0.9577 on benchmark datasets, showcasing excellent accuracy and performance in predicting circRNA–RBP interactions for both full‐length sequences and fragments [253]. Moreover, the recently introduced CoPRA model, developed through a multi‐institutional consortium, has gained significant attention for its innovative integration of protein language models and RNA language models within a sophisticated architecture designed for protein–RNA binding affinity prediction. Benchmark evaluations show that CoPRA achieves state‐of‐the‐art performance across various datasets, positioning it as a promising framework for future investigations into circRNA–RBP interactions [254].

## 9 Challenges in the Clinical Translation of CircRNAs

### 9.1 Challenges in the Standardized Study of CircRNAs

While the rapid advancement of high‐throughput sequencing technologies and algorithmic innovations has significantly accelerated circRNA research, these developments also introduce challenges in establishing standardized experimental and analytical frameworks. At the library preparation stage, there is considerable divergence between RNase R‐treated and untreated datasets. Critical digestion conditions, such as enzyme concentration, experimental time, and reaction temperature, significantly impact the results [255]. Additionally, specific RNA structures, including G‐quadruplexes, histone mRNAs, and small nuclear RNAs, exhibit intrinsic resistance to RNase R digestion [256, 257]. Furthermore, despite the availability of numerous computational algorithms for circRNA detection, there are substantial variations in sensitivity across different methods. A 2023 benchmarking study comparing 16 distinct algorithms revealed identification discrepancies ranging from 1372 to 58,032 circRNAs per algorithm within identical cell lines [256]. Consequently, integrating multiple algorithmic tools is essential. CircComPara2 incorporates up to eight circRNA detection tools alongside various analytical modules [258]; however, such multitool integration has not yet established itself as a universal gold‐standard computational framework, necessitating comprehensive benchmarking studies and algorithmic innovations.

Inherent differences in sequencing technologies also pose significant challenges for circRNA normalization. Conventional short‐read sequencing (second‐generation sequencing) generates reads typically ranging from 50 to 300 nucleotides in length [259], enabling high‐throughput data acquisition. However, these short reads are generally incapable of spanning the full BSJ, necessitating reliance on split‐alignment strategies to identify discontinuous sequences flanking the BSJ. This approach is associated with higher false‐positive rates and a dependency on existing reference genome annotations [260]. In contrast, long‐read sequencing (third‐generation sequencing) offers distinct advantages in detecting full‐length circRNAs and characterizing isoforms. This technology can directly span entire circRNA molecules to capture complete sequences, thereby enabling the detection of a greater number of alternative circularization events and internal alternative splicing events. However, long‐read sequencing faces technical limitations such as lower base‐call accuracy and read truncation, which may compromise the quantification of splice isoforms [261]. Additionally, the high costs associated with these technologies present challenges in standardizing research protocols.

Recent advancements address these issues through Longcell, an analytical pipeline developed at Stanford University School of Medicine. This framework incorporates four core technological innovations that achieve Spearman correlation coefficients exceeding 0.85 for single‐cell isoform quantification accuracy using nanopore long‐read data, positioning it as an indispensable analytical tool for long‐read sequencing applications [262]. Finally, discrepancies, redundancies, and inconsistencies in circRNA annotations (including IDs, sequences, genomic locations, host genes, and expression profiles) across public databases, coupled with the absence of standardized nomenclature, result in multiple designations for identical circRNAs, thus posing significant challenges for researchers integrating and cross‐referencing results from diverse sources.

### 9.2 Ethical Issues in the Clinical Translational Research of CircRNA

Clinical translational research serves as a crucial bridge between fundamental medical discoveries and front‐line clinical practice. Current efforts in circRNA translation primarily focus on diagnostic biomarkers, cancer immunotherapy, drug development, and innovative vaccine design. However, the field is still in its infancy, with most circRNA‐related studies remaining at the preclinical stage [74, 116, 263]. Consequently, there is an urgent need for standardized ethical frameworks to address the associated moral and regulatory challenges.

The most prominent application of circRNAs in clinical translation is in disease diagnosis and biomarker development. Due to their structural stability and abundance in peripheral blood, urine, and exosomes, circRNAs have been extensively studied as noninvasive biomarkers for gastrointestinal cancers, urological tumors, and other diseases [264, 265]. Nevertheless, privacy and data security have become critical concerns in the clinical implementation of circRNA‐based biomarkers, raising ethical issues about protecting sensitive patient information from unauthorized access and misuse [266, 267]. Therefore, obtaining informed consent before collecting biospecimens and associated data, such as pathological tissues, plasma, and urine, is essential. For biomarker research, the informed consent process should involve thorough consultations between patients and relevant institutions, such as hospitals and laboratories, rather than just a signature on a form. Given the rapidly evolving nature of circRNA research and the potential for future secondary use of samples, adopting a dynamic consent framework may better safeguard patient rights and autonomy [268]. Furthermore, the clinical translation of circRNAs in cancer immunotherapy and drug development faces substantial ethical and regulatory challenges, largely due to differing approval pathways across jurisdictions. In the United States, biological products require premarket approval from the United States Food and Drug Administration (US FDA). In Europe, advanced therapy medicinal products, including gene‐, tissue‐, and cell‐based therapies, are regulated under regulation (EC) No. 1394/2007 and must obtain centralized marketing authorization from the European Medicines Agency [269]. In China, the National Medical Products Administration oversees the development, production, distribution, and use of pharmaceuticals, including biological drugs [270]. Navigating innovative therapies through these diverse regulatory frameworks presents significant challenges, necessitating continuous adaptation through sustained dialogue with all stakeholders.

Moreover, due to the unique survival characteristics of cancer patients, most current circRNA‐based therapeutic regimens have yet to demonstrate improvements in long‐term survival, leaving considerable uncertainty regarding their enduring benefits and risks. To ensure participant safety, it is essential to establish unified clinical translation protocols through multinational, multicenter collaborations [271].

## 10 Conclusions and Perspective

Initially, circRNAs were considered mere “noise” in the translation process. However, with technological advancements, circRNAs have been found to have multiple functions, including acting as miRNA sponges, regulating parental gene transcription, and serving as protein translation templates [272]. These diverse functions are associated with various physiological and pathological processes. Due to their unique structural characteristics, circRNAs exhibit stable in vivo expression and tissue specificity, making them potential biomarkers [101, 273].

Elucidating the detailed biological functions of circRNAs requires high‐precision spatial mapping of their subcellular localization. Super‐resolution microscopy techniques, such as stochastic optical reconstruction microscopy and photoactivated localization microscopy, have become the primary methodologies for subcellular circRNA localization and functional characterization [274, 275]. However, super‐resolution imaging demands sophisticated instrumentation and meticulous calibration, leading to significant economic and temporal costs. This highlights the need for technological refinements and cost‐containment strategies. Multimodal imaging, which integrates optical microscopy with mass spectrometry imaging, offers a promising approach by enabling concurrent mapping of circRNA spatial localization and interactions, thus providing comprehensive insights into biological processes from molecular to tissue levels. Importantly, these techniques generate vast datasets alongside high‐precision images, necessitating the development of advanced machine and deep learning algorithms to optimize data processing, automate analysis pipelines, and enable real‐time interpretation of imaging data.

Given their superior structural integrity compared with mRNAs and their pleiotropic functional modalities in vivo, circRNAs are promising candidates for engineered therapeutic vectors. Although current technologies can synthesize both short and long circRNA sequences, previous studies have shown that RNAs generated through different circularization methods can elicit markedly distinct immune responses in humans [16, 276]. This underscores the need for context‐specific circRNA synthesis platforms to ensure predictable outcomes across diverse biological applications. Additionally, circRNA delivery poses significant challenges. While LNP systems are established delivery platforms, their encapsulation efficiency for circRNAs is substantially lower than for linear mRNAs due to the larger molecular size of circular transcripts. Multiple biotechnology firms are optimizing LNP formulations to enhance delivery efficacy [277]. Targeted delivery is another critical hurdle, as the inherent hepatotropism of LNPs limits therapeutic applications to hepatic tissues, whereas most diseases require delivery to extrahepatic organs [278]. Consequently, developing exosomes, which are naturally evolved intercellular communication vehicles, as delivery vectors represents a compelling alternative. Several companies are now focusing on exosome‐based delivery technologies, despite the engineering complexities and substantial manufacturing costs.

Over the past decade, AI has emerged as a powerful tool in biomedical research, particularly with the advent of large language models like ChatGPT and Claude, alongside advanced deep learning algorithms. Conventional approaches for screening and validating therapeutic circRNA candidates typically require months to years, whereas AI‐driven pipelines enhance efficiency by over 50%. Chinese research teams have made substantial advancements in circRNA vaccine development through AI‐driven multimodal optimization [279]. Future efforts necessitate expanded translational investigations into circRNA‐based therapeutics across diverse oncological contexts. Integrating multiomics data, including single‐cell sequencing, epigenomic profiles, and protein interaction networks, through AI frameworks enables robust prediction of circRNA targets with therapeutic potential. However, fragmented medical data across isolated databases and stringent privacy constraints create significant data barriers, impeding AI model development due to prohibitive training costs. Establishing curated, publicly accessible multiomics repositories spanning diverse diseases is therefore imperative. Furthermore, the opaque decision‐making processes of deep neural networks necessitate rigorous validation of model interpretability through SHapley Additive exPlanations frameworks and experimental verification.

Collectively, circRNAs demonstrate superior structural and functional attributes in oncology research, driving a paradigm shift from traditional biomarkers, anticancer therapies, and mRNA vaccines toward multimodal circRNA‐based therapeutics. In Q4 2024, the US FDA granted Investigational New Drug (IND) clearance for the Phase I/IIa RXRG001 trial (SPRINX‐1 study), representing the first US FDA‐approved circRNA therapy globally. Furthermore, China's Center for Drug Evaluation (CDE) accepted the IND application for BioCrown's circRNA drug TI‐0093 in June 2025. This investigational injection targets HPV16‐positive advanced recurrent or metastatic solid tumors, constituting the only circRNA antitumor agent in IND‐stage development. Sustained research investment and substantial funding remain imperative to ensure translational feasibility.

## Author Contributions

Zehao Ding, Zai Luo, and Liao Zhang conceptualized the manuscript, performed the literature search, wrote the original draft, and revised and edited the manuscript. Shaopeng Zhang revised and edited the manuscript. Renchao Zhang, Zhengjun Qiu, and Chen Huang supervised, revised, and edited the manuscript. All authors read and approved the final manuscript.

## Funding Information

CH is supported by the National Natural Science Foundation of China (No.82472921 and No. 82072662), Key Projects of the Interdisciplinary Program of Medicine and Engineering at Shanghai Jiao Tong University (No. 24×010301419), and the 2021 Shanghai ‘Rising Stars of Medical Talent’ Youth Development Program: Outstanding Youth Medical Talents. ZL is sponsored by Shanghai Rising‐Star Program Sailing Program (No. 24YF2734700) and Young Scientists Fund of the National Natural Science Foundation of China (Grant No. 82504100).

## Ethics Statement

The authors have nothing to report.

## Conflicts of Interest

The authors declare no conflicts of interest.

## Data Availability

The authors have nothing to report.

## References

[1] W. Li , J.‐Q. Liu , M. Chen , et al., “Circular RNA in Cancer Development and Immune Regulation,” Journal of Cellular and Molecular Medicine 26, no. 6 (2020): 1785–1798. 33277969 10.1111/jcmm.16102 PMC8918416

[2] D. Kolakofsky , “Isolation and Characterization of sendai Virus DI‐RNAs,” Cell 8, no. 4 (1976): 547–555. 182384 10.1016/0092-8674(76)90223-3

[3] B. P. Nicolet , S. Engels , F. Aglialoro , V. D. Akker E , V. Lindern M , and M. C. Wolkers , “Circular RNA Expression in human Hematopoietic Cells Is Widespread and Cell‐type Specific,” Nucleic Acids Research 46, no. 16 (2018): 8168–8180. 30124921 10.1093/nar/gky721 PMC6144802

[4] K. Nemeth , R. Bayraktar , M. Ferracin , et al., “Non‐coding RNAs in Disease: From Mechanisms to Therapeutics,” Nature Reviews Genetics 25, no. 3 (2023): 211–232. 10.1038/s41576-023-00662-1 37968332

[5] I. B. Filippenkov , E. O. Kalinichenko , S. A. Limborska , and L. V. Dergunova , “Circular RNAs‐one of the Enigmas of the Brain,” Neurogenetics 18, no. 1 (2016): 1–6. 27449796 10.1007/s10048-016-0490-4

[6] G. Huang , M. Liang , H. Liu , et al., “CircRNA hsa_circRNA_104348 Promotes Hepatocellular Carcinoma Progression Through Modulating miR‐187‐3p/RTKN2 Axis and Activating Wnt/β‐catenin Pathway,” Cell Death & Disease 11, no. 12 (2020): 1065. 33311442 10.1038/s41419-020-03276-1 PMC7734058

[7] F. Yang , Q. Ma , B. Huang , et al., “CircNFATC3 promotes the Proliferation of Gastric Cancer Through Binding to IGF2BP3 and Restricting Its Ubiquitination to Enhance CCND1 mRNA Stability,” Journal of Translational Medicine 21, no. 1 (2023): 402. 37340423 10.1186/s12967-023-04235-y PMC10280940

[8] B. Fu , W. Liu , C. Zhu , et al., “Circular RNA circBCBM1 Promotes Breast Cancer Brain Metastasis by Modulating miR‐125a/BRD4 Axis,” International Journal of Biological Sciences 17, no. 12 (2021): 3104–3117. 34421353 10.7150/ijbs.58916 PMC8375234

[9] X. Yu , W. Xiao , H. Song , Y. Jin , J. Xu , and X. Liu , “CircRNA_100876 sponges miR‐136 to Promote Proliferation and Metastasis of Gastric Cancer by Upregulating MIEN1 Expression,” Gene 748 (2020): 144678. 32305633 10.1016/j.gene.2020.144678

[10] C. Wu , S. Wang , T. Cao , et al., “Newly Discovered Mechanisms That Mediate Tumorigenesis and Tumour Progression: CircRNA‐encoded Proteins,” Journal of Cellular and Molecular Medicine 27, no. 12 (2023): 1609–1620. 37070530 10.1111/jcmm.17751 PMC10273065

[11] P. Wu , Y. Mo , M. Peng , et al., “Emerging Role of Tumor‐related Functional Peptides Encoded by lncRNA and circRNA,” Molecular Cancer 19, no. 1 (2020): 22. 32019587 10.1186/s12943-020-1147-3 PMC6998289

[12] Y. Tang , F. Yuan , M. Cao , et al., “CircRNA‐mTOR Promotes Hepatocellular Carcinoma Progression and Lenvatinib Resistance Through the PSIP1/c‐myc Axis,” Advanced Science (Weinheim, Baden‐Wurttemberg, Germany) 12, no. 20 (2025): e2410591. 40231634 10.1002/advs.202410591 PMC12120768

[13] J. Zhang , Q. Yu , W. Zhu , et al., “Recent Advances in the Role of circRNA in Cisplatin Resistance in Tumors,” Cancer Gene Therapy 32, no. 5 (2025): 497–506. 40148680 10.1038/s41417-025-00899-4

[14] J. Yi , J. Du , X. Chen , et al., “A circRNA‐mRNA Pairing Mechanism Regulates Tumor Growth and Endocrine Therapy Resistance in ER‐positive Breast Cancer,” Proceedings of the National Academy of Sciences of the United States of America 122, no. 8 (2025): e2420383122. 40233410 10.1073/pnas.2420383122 PMC11874584

[15] J. Wan , C. Wang , Z. Wang , et al., “CXCL13 promotes Broad Immune Responses Induced by Circular RNA Vaccines,” Proceedings of the National Academy of Sciences of the United States of America 121, no. 44 (2024): e2406434121. 39436660 10.1073/pnas.2406434121 PMC11536096

[16] Y. Alshehry , X. Liu , Y. Zhang , et al., “Investigation of the Impact of Lipid Nanoparticle Compositions on the Delivery and T Cell Response of circRNA Vaccine,” Journal of Controlled Release: Official Journal of the Controlled Release Society 381 (2025): 113617. 40107513 10.1016/j.jconrel.2025.113617 PMC11994274

[17] J. Xu , Y. Liu , J. Zhang , et al., “Single‐droplet Dual‐target Quantification of circRNA Biomarkers for Colorectal Cancer Screening,” Advanced Science (Weinheim, Baden‐Wurttemberg, Germany) 12, no. 38 (2025): e06159. 40583153 10.1002/advs.202506159 PMC12520571

[18] Y. Sheng , L. Yang , B. Wang , et al., “Plasma‐derived circALG8 and circCAMTA1 as a Panel for Early Diagnosis of Non‐small Cell Lung Cancer,” Biomarkers in Medicine 19, no. 16 (2025): 725–736. 40754803 10.1080/17520363.2025.2542115 PMC12416200

[19] Y. Yu , W. Zheng , C. Ji , et al., “Tumor‐derived circRNAs as Circulating Biomarkers for Breast Cancer,” Frontiers in Pharmacology 13 (2022): 811856. 35242035 10.3389/fphar.2022.811856 PMC8886293

[20] A. M. Filho , M. Laversanne , J. Ferlay , et al., “The GLOBOCAN 2022 Cancer Estimates: Data Sources, Methods, and a Snapshot of the Cancer Burden Worldwide,” International Journal of Cancer 156, no. 7 (2024): 1336–1346. 39688499 10.1002/ijc.35278

[21] W. R. Jeck , J. A. Sorrentino , K. Wang , et al., “Circular RNAs Are Abundant, Conserved, and Associated With ALU Repeats,” Rna 19, no. 2 (2013): 141–157. 23249747 10.1261/rna.035667.112 PMC3543092

[22] S. Memczak , M. Jens , A. Elefsinioti , et al., “Circular RNAs Are a Large Class of Animal RNAs With Regulatory Potency,” Nature 495, no. 7441 (2013): 333–338. 23446348 10.1038/nature11928

[23] I. Legnini , G. Di Timoteo , F. Rossi , et al., “Circ‐ZNF609 Is a Circular RNA That Can be Translated and Functions in Myogenesis,” Molecular Cell 66, no. 1 (2017): 22–37.e9. 28344082 10.1016/j.molcel.2017.02.017 PMC5387670

[24] N. R. Pamudurti , O. Bartok , M. Jens , et al., “Translation of CircRNAs,” Molecular Cell 66, no. 1 (2017): 9–21.e7. 28344080 10.1016/j.molcel.2017.02.021 PMC5387669

[25] L. Qu , Z. Yi , Y. Shen , et al., “Circular RNA Vaccines Against SARS‐CoV‐2 and Emerging Variants,” Cell 185, no. 10 (2022): 1728–1744.e16. 35460644 10.1016/j.cell.2022.03.044 PMC8971115

[26] G. Pisignano , D. C. Michael , T. H. Visal , R. Pirlog , M. Ladomery , and G. A. Calin , “Going Circular: History, Present, and Future of circRNAs in Cancer,” Oncogene 42, no. 38 (2023): 2783–2800. 37587333 10.1038/s41388-023-02780-w PMC10504067

[27] L.‐L. Chen , “The Expanding Regulatory Mechanisms and Cellular Functions of Circular RNAs,” Nature Reviews Molecular Cell Biology 21, no. 8 (2020): 475–490. 32366901 10.1038/s41580-020-0243-y

[28] V. Singh , M. H. Uddin , J. A. Zonder , A. S. Azmi , and S. K. Balasubramanian , “Circular RNAs in Acute Myeloid Leukemia,” Molecular Cancer 20, no. 1 (2021): 149. 34794438 10.1186/s12943-021-01446-z PMC8600814

[29] I. Trsova , A. Hrustincova , Z. Krejcik , et al., “Expression of Circular RNAs in Myelodysplastic Neoplasms and Their Association With Mutations in the Splicing Factor Gene SF3B1,” Molecular Oncology 17, no. 12 (2023): 2565–2583. 37408496 10.1002/1878-0261.13486 PMC10701770

[30] D. Inoue , G.‐L. Chew , B. Liu , et al., “Spliceosomal Disruption of the Non‐canonical BAF Complex in Cancer,” Nature 574, no. 7778 (2019): 432–436. 31597964 10.1038/s41586-019-1646-9 PMC6858563

[31] L. S. Kristensen , M. S. Andersen , L. V. W. Stagsted , K. K. Ebbesen , T. B. Hansen , and J. Kjems , “The Biogenesis, Biology and Characterization of Circular RNAs,” Nature Reviews Genetics 20, no. 11 (2019): 675–691. 10.1038/s41576-019-0158-7 31395983

[32] K. K. Ebbesen , J. Kjems , and T. B. Hansen , “Circular RNAs: Identification, Biogenesis and Function,” Biochimica Et Biophysica Acta 1859, no. 1 (2016): 163–168. 26171810 10.1016/j.bbagrm.2015.07.007

[33] L. Li , C. Wei , Y. Xie , et al., “Expanded Insights Into the Mechanisms of RNA‐binding Protein Regulation of circRNA Generation and Function in Cancer Biology and Therapy,” Genes & Diseases 12, no. 4 (2025): 101383. 40290118 10.1016/j.gendis.2024.101383 PMC12022641

[34] C. Shan , Y. Zhang , X. Hao , et al., “Biogenesis, Functions and Clinical Significance of circRNAs in Gastric Cancer,” Molecular Cancer 18, no. 1 (2019): 136. 31519189 10.1186/s12943-019-1069-0 PMC6743094

[35] P. Montañés‐Agudo , V. D. Made , S. Aufiero , A. J. Tijsen , Y. M. Pinto , and E. E. Creemers , “Quaking Regulates Circular RNA Production in Cardiomyocytes,” Journal of Cell Science 136, no. 13 (2023): jcs261120. 37272356 10.1242/jcs.261120 PMC10323251

[36] D. Cao , “An Autoregulation Loop in fust‐1 for Circular RNA Regulation in caenorhabditis elegans,” Genetics 219, no. 3 (2021): iyab145. 34740247 10.1093/genetics/iyab145 PMC8570788

[37] R. Ashwal‐Fluss , M. Meyer , N. R. Pamudurti , et al., “circRNA Biogenesis Competes With Pre‐mRNA Splicing,” Molecular Cell 56, no. 1 (2014): 55–66. 25242144 10.1016/j.molcel.2014.08.019

[38] E. Anastasiadou , L. S. Jacob , and F. J. Slack , “Non‐coding RNA Networks in Cancer,” Nature Reviews Cancer 18, no. 1 (2018): 5–18. 29170536 10.1038/nrc.2017.99 PMC6337726

[39] J. Ma , W. W. Du , K. Zeng , et al., “An Antisense Circular RNA circSCRIB Enhances Cancer Progression by Suppressing Parental Gene Splicing and Translation,” Molecular Therapy 29, no. 9 (2021): 2754–2768. 34365033 10.1016/j.ymthe.2021.08.002 PMC8417507

[40] J. M. Burke , A. R. Gilchrist , S. L. Sawyer , and R. Parker , “RNase L Limits Host and Viral Protein Synthesis via Inhibition of mRNA Export,” Science Advances 7, no. 23 (2021): eabh2479. 34088676 10.1126/sciadv.abh2479 PMC8177694

[41] C.‐X. Liu , X. Li , F. Nan , et al., “Structure and Degradation of Circular RNAs Regulate PKR Activation in Innate Immunity,” Cell 177, no. 4 (2019): 865–880. e21. 31031002 10.1016/j.cell.2019.03.046

[42] L. Ren , Q. Jiang , L. Mo , et al., “Mechanisms of Circular RNA Degradation,” Communications Biology 5, no. 1 (2022): 1355. 36494488 10.1038/s42003-022-04262-3 PMC9734648

[43] M. Saramago , P. J. D. Costa , S. C. Viegas , and C. M. Arraiano , “The Implication of mRNA Degradation Disorders on human DISease: Focus on DIS3 and DIS3‐Like Enzymes,” Advances in Experimental Medicine and Biology 1157 (2019): 85–98. 31342438 10.1007/978-3-030-19966-1_4

[44] J. W. Fischer , V. F. Busa , Y. Shao , and A. K. L. Leung , “Structure‐mediated RNA Decay by UPF1 and G3BP1,” Molecular Cell 78, no. 1 (2020): 70–84. 32017897 10.1016/j.molcel.2020.01.021 PMC8055448

[45] X. Tao , S.‐N. Zhai , C.‐X. Liu , et al., “Degradation of Circular RNA by the Ribonuclease DIS3,” Molecular Cell 85, no. 8 (2025): 1674–1685. 39965568 10.1016/j.molcel.2025.01.012

[46] Z. Pan , G.‐F. Li , M.‐L. Sun , et al., “MicroRNA‐1224 Splicing CircularRNA‐Filip1l in an Ago2‐dependent Manner Regulates Chronic Inflammatory Pain via Targeting Ubr5,” The Journal of Neuroscience: The Official Journal of the Society for Neuroscience 39, no. 11 (2019): 2125–2143. 30651325 10.1523/JNEUROSCI.1631-18.2018 PMC6507086

[47] T. B. Hansen , E. D. Wiklund , J. B. Bramsen , et al., “miRNA‐dependent Gene Silencing Involving Ago2‐mediated Cleavage of a Circular Antisense RNA,” The EMBO Journal 30, no. 21 (2011): 4414–4422. 21964070 10.1038/emboj.2011.359 PMC3230379

[48] R. Jia , M.‐S. Xiao , Z. Li , G. Shan , and C. Huang , “Defining an Evolutionarily Conserved Role of GW182 in Circular RNA Degradation,” Cell Discovery 5 (2019): 45. 31636958 10.1038/s41421-019-0113-y PMC6796862

[49] Y. Guo , Y. Guo , C. Chen , et al., “Circ3823 contributes to Growth, Metastasis and Angiogenesis of Colorectal Cancer: Involvement of miR‐30c‐5p/TCF7 Axis,” Molecular Cancer 20, no. 1 (2021): 93. 34172072 10.1186/s12943-021-01372-0 PMC8229759

[50] S. Xiao , S. Duan , M. A. Caligiuri , et al., “YTHDF2: A Key RNA Reader and Antitumor Target,” Trends in Immunology 46, no. 6 (2025): 485–498. 40399203 10.1016/j.it.2025.04.003 PMC13715896

[51] O. H. Park , H. Ha , Y. Lee , et al., “Endoribonucleolytic Cleavage of m6A‐containing RNAs by RNase P/MRP Complex,” Molecular Cell 74, no. 3 (2019): 494–507. 30930054 10.1016/j.molcel.2019.02.034

[52] H. Du , Y. Zhao , J. He , et al., “YTHDF2 destabilizes M(6)a‐containing RNA Through Direct Recruitment of the CCR4‐NOT Deadenylase Complex,” Nature Communications 7 (2016): 12626. 10.1038/ncomms12626 PMC5007331 27558897

[53] M. Hetzer , O. J. Gruss , and I. W. Mattaj , “The Ran GTPase as a Marker of Chromosome Position in Spindle Formation and Nuclear Envelope Assembly,” Nature Cell Biology 4, no. 7 (2002): E177–E184. 12105431 10.1038/ncb0702-e177

[54] L. H. Ngo , A. G. Bert , B. K. Dredge , et al., “Nuclear Export of Circular RNA,” Nature 627, no. 8002 (2024): 212–220. 38355801 10.1038/s41586-024-07060-5

[55] Y. Zhan , J. Du , Z. Min , et al., “Carcinoma‐associated Fibroblasts Derived Exosomes Modulate Breast Cancer Cell Stemness Through Exonic circHIF1A by miR‐580‐5p in Hypoxic Stress,” Cell Death Discovery 7, no. 1 (2021): 141. 34120145 10.1038/s41420-021-00506-z PMC8197761

[56] H. Yang , H. Zhang , Y. Yang , et al., “Hypoxia Induced Exosomal circRNA Promotes Metastasis of Colorectal Cancer via Targeting GEF‐H1/RhoA Axis,” Theranostics 10, no. 18 (2020): 8211–8226. 32724467 10.7150/thno.44419 PMC7381736

[57] R. Kalluri and V. S. LeBleu , “The Biology, Function, and Biomedical Applications of Exosomes,” Science 367, no. 6478 (2020): eaau6977. 32029601 10.1126/science.aau6977 PMC7717626

[58] X. Zhang , Y. Xu , L. Ma , et al., “Essential Roles of Exosome and circRNA_101093 on Ferroptosis Desensitization in Lung Adenocarcinoma,” Cancer Communications 42, no. 4 (2022): 287–313. 35184419 10.1002/cac2.12275 PMC9017758

[59] C. Deng , M. Huo , H. Chu , et al., “Exosome circATP8A1 Induces Macrophage M2 Polarization by Regulating the miR‐1‐3p/STAT6 Axis to Promote Gastric Cancer Progression,” Molecular Cancer 23, no. 1 (2024): 49. 38459596 10.1186/s12943-024-01966-4 PMC10921793

[60] H. Xie , J. Yao , Y. Wang , et al., “Exosome‐transmitted circVMP1 Facilitates the Progression and Cisplatin Resistance of Non‐small Cell Lung Cancer by Targeting miR‐524‐5p‐METTL3/SOX2 Axis,” Drug Delivery 29, no. 1 (2022): 1257–1271. 35467477 10.1080/10717544.2022.2057617 PMC9045767

[61] M. Xie , T. Yu , X. Jing , et al., “Exosomal circSHKBP1 Promotes Gastric Cancer Progression via Regulating the miR‐582‐3p/HUR/VEGF Axis and Suppressing HSP90 Degradation,” Molecular Cancer 19, no. 1 (2020): 112. 32600329 10.1186/s12943-020-01208-3 PMC7322843

[62] T. He , Q. Zhang , P. Xu , et al., “Extracellular Vesicle‐circEHD2 Promotes the Progression of Renal Cell Carcinoma by Activating Cancer‐associated Fibroblasts,” Molecular Cancer 22, no. 1 (2023): 117. 37481520 10.1186/s12943-023-01824-9 PMC10362694

[63] L.‐L. Yu , Q. Xiao , B. Yu , et al., “CircRNAs in Tumor Immunity and Immunotherapy: Perspectives From Innate and Adaptive Immunity,” Cancer Letters 564 (2023): 216219. 37146937 10.1016/j.canlet.2023.216219

[64] Y. Enuka , M. Lauriola , M. E. Feldman , et al., “Circular RNAs Are Long‐lived and Display Only Minimal Early Alterations in Response to a Growth Factor,” Nucleic Acids Research 44, no. 3 (2015): 1370–1383. 26657629 10.1093/nar/gkv1367 PMC4756822

[65] R.‐X. Chen , X. Chen , L.‐P. Xia , et al., “N6‐methyladenosine Modification of circNSUN2 Facilitates Cytoplasmic Export and Stabilizes HMGA2 to Promote Colorectal Liver Metastasis,” Nature Communications 10, no. 1 (2019): 4695. 10.1038/s41467-019-12651-2 PMC6795808 31619685

[66] C. Xu , E. Jun , Y. Okugawa , et al., “A Circulating Panel of circRNA Biomarkers for the Noninvasive and Early Detection of Pancreatic Ductal Adenocarcinoma,” Gastroenterology 166, no. 1 (2024): 178–190.e16. 37839499 10.1053/j.gastro.2023.09.050 PMC10843014

[67] S. Misir , N. Wu , and B. B. Yang , “Specific Expression and Functions of Circular RNAs,” Cell Death & Differentiation 29, no. 3 (2022): 481–491. 35169296 10.1038/s41418-022-00948-7 PMC8901656

[68] S. Werfel , S. Nothjunge , T. Schwarzmayr , et al., “Characterization of Circular RNAs in human, Mouse and Rat Hearts,” Journal of Molecular and Cellular Cardiology 98 (2016): 103–107. 27476877 10.1016/j.yjmcc.2016.07.007

[69] D. Huang , X. Zhu , S. Ye , et al., “Tumour Circular RNAs Elicit Anti‐tumour Immunity by Encoding Cryptic Peptides,” Nature 625, no. 7995 (2023): 593–602. 38093017 10.1038/s41586-023-06834-7

[70] J. Ge , Y. Meng , J. Guo , et al., “Human Papillomavirus‐encoded Circular RNA circE7 Promotes Immune Evasion in Head and Neck Squamous Cell Carcinoma,” Nature Communications 15, no. 1 (2024): 8609. 10.1038/s41467-024-52981-4 PMC11452643 39366979

[71] R. Song , S. Ma , J. Xu , et al., “A Novel Polypeptide Encoded by the Circular RNA ZKSCAN1 Suppresses HCC via Degradation of mTOR,” Molecular Cancer 22, no. 1 (2023): 16. 36691031 10.1186/s12943-023-01719-9 PMC9869513

[72] H. Luo , Y. Zhu , J. Wang , et al., “Comprehensive Profile and Contrastive Analysis of Circular RNA Expression in Cervical Squamous Carcinoma and Adenocarcinoma,” PeerJ 11 (2023): e14759. 36721776 10.7717/peerj.14759 PMC9884480

[73] G. Li , X. Xu , L. Yang , et al., “Exploring the Association Between circRNA Expression and Pediatric Obesity Based on a Case‐control Study and Related Bioinformatics Analysis,” BMC Pediatrics 23, no. 1 (2023): 561. 37957626 10.1186/s12887-023-04261-1 PMC10642011

[74] M. Teng , J. Guo , X. Xu , et al., “Circular RMST Cooperates With Lineage‐driving Transcription Factors to Govern Neuroendocrine Transdifferentiation,” Cancer Cell 43, no. 5 (2025): 891–904.e10. 40250444 10.1016/j.ccell.2025.03.027 PMC12991876

[75] Z. Pan , R. Zhao , B. Li , et al., “EWSR1‐induced circNEIL3 Promotes Glioma Progression and Exosome‐mediated Macrophage Immunosuppressive Polarization via Stabilizing IGF2BP3,” Molecular Cancer 21, no. 1 (2022): 16. 35031058 10.1186/s12943-021-01485-6 PMC8759291

[76] L. S. Kristensen , K. K. Ebbesen , M. Sokol , et al., “Spatial Expression Analyses of the Putative Oncogene ciRS‐7 in Cancer Reshape the microRNA Sponge Theory,” Nature Communications 11, no. 1 (2020): 4551. 10.1038/s41467-020-18355-2 PMC7486402 32917870

[77] J. Li , W. Zhou , H. Wang , et al., “Exosomal Circular RNAs in Tumor Microenvironment: An Emphasis on Signaling Pathways and Clinical Opportunities,” MedComm 5, no. 12 (2024): e70019. 39584047 10.1002/mco2.70019 PMC11586091

[78] P.‐F. Zhang , C. Gao , X.‐Y. Huang , et al., “Cancer Cell‐derived Exosomal circUHRF1 Induces Natural Killer Cell Exhaustion and May Cause Resistance to Anti‐PD1 Therapy in Hepatocellular Carcinoma,” Molecular Cancer 19, no. 1 (2020): 110. 32593303 10.1186/s12943-020-01222-5 PMC7320583

[79] C. Chen , Y. Liu , L. Liu , et al., “Exosomal circTUBGCP4 Promotes Vascular Endothelial Cell Tipping and Colorectal Cancer Metastasis by Activating Akt Signaling Pathway,” Journal of Experimental & Clinical Cancer Research 42, no. 1 (2023): 46. 36793126 10.1186/s13046-023-02619-y PMC9930311

[80] D. P. Bartel , “MicroRNAs: Genomics, Biogenesis, Mechanism, and Function,” Cell 116, no. 2 (2004): 281–297. 14744438 10.1016/s0092-8674(04)00045-5

[81] X. Zhang , S. Wang , H. Wang , et al., “Circular RNA circNRIP1 Acts as a microRNA‐149‐5p Sponge to Promote Gastric Cancer Progression via the AKT1/mTOR Pathway,” Molecular Cancer 18, no. 1 (2019): 20. 30717751 10.1186/s12943-018-0935-5 PMC6360801

[82] D.‐L. Chen , H. Sheng , D.‐S. Zhang , et al., “The Circular RNA circDLG1 Promotes Gastric Cancer Progression and Anti‐PD‐1 Resistance Through the Regulation of CXCL12 by Sponging miR‐141‐3p,” Molecular Cancer 20, no. 1 (2021): 166. 34911533 10.1186/s12943-021-01475-8 PMC8672580

[83] H. Li , B. Su , Y. Jiang , et al., “Circular RNA circDCUN1D4 Suppresses Hepatocellular Carcinoma Development via Targeting the miR‐590‐5p/TIMP3 Axis,” Molecular Cancer 24, no. 1 (2025): 95. 40128740 10.1186/s12943-025-02300-2 PMC11934760

[84] J. Zhu , Q. Li , Z. Wu , et al., “Circular RNA‐mediated miRNA Sponge & RNA Binding Protein in Biological Modulation of Breast Cancer,” Non‐coding RNA Research 9, no. 1 (2024): 262–276. 38282696 10.1016/j.ncrna.2023.12.005 PMC10818160

[85] Y. Wei , X. Chen , C. Liang , et al., “A Noncoding Regulatory RNAs Network Driven by Circ‐CDYL Acts Specifically in the Early Stages Hepatocellular Carcinoma,” Hepatology 71, no. 1 (2020): 130–147. 31148183 10.1002/hep.30795

[86] W. Du , X. Quan , C. Wang , et al., “Regulation of Tumor Metastasis and CD8+ T Cells Infiltration by circRNF216/miR‐576‐5p/ZC3H12C Axis in Colorectal Cancer,” Cellular & Molecular Biology Letters 29, no. 1 (2024): 19. 38267865 10.1186/s11658-024-00539-z PMC10809481

[87] Z. Luo , Z. Rong , J. Zhang , et al., “Circular RNA circCCDC9 Acts as a miR‐6792‐3p Sponge to Suppress the Progression of Gastric Cancer Through Regulating CAV1 Expression,” Molecular Cancer 19, no. 1 (2020): 86. 32386516 10.1186/s12943-020-01203-8 PMC7210689

[88] S. He , E. Valkov , S. Cheloufi , et al., “The Nexus Between RNA‐binding Proteins and Their Effectors,” Nature Reviews Genetics 24, no. 5 (2023): 276–294. 10.1038/s41576-022-00550-0 PMC10714665 36418462

[89] J.‐Y. Liao , B. Yang , C.‐P. Shi , et al., “RBPWorld for Exploring Functions and Disease Associations of RNA‐binding Proteins Across Species,” Nucleic Acids Research 53 (2024): D220–D232. 10.1093/nar/gkae1028 PMC11701580 39498484

[90] X. Hu , G. Chen , Y. Huang , et al., “Integrated Multiomics Reveals Silencing of has_circ_0006646 Promotes TRIM21‐mediated NCL Ubiquitination to Inhibit Hepatocellular Carcinoma Metastasis,” Advanced Science 11, no. 16 (2024): e2306915. 38357830 10.1002/advs.202306915 PMC11040345

[91] R.‐G. Chen , G.‐R. Chen , X.‐X. Jiang , et al., “circ0006646 serves as a Robust Prognostic Biomarker for Post‐transplant Tumor Recurrence and Survival in Hepatocellular Carcinoma Patients,” Hepatobiliary & Pancreatic Diseases International 24, no. 6 (2025): 616–624. 40701899 10.1016/j.hbpd.2025.07.002

[92] Z. Wang , A. Sun , A. Yan , et al., “Circular RNA MTCL1 Promotes Advanced Laryngeal Squamous Cell Carcinoma Progression by Inhibiting C1QBP Ubiquitin Degradation and Mediating Beta‐catenin Activation,” Molecular Cancer 21, no. 1 (2022): 92. 35366893 10.1186/s12943-022-01570-4 PMC8976408

[93] F. Yang , A. Hu , D. Li , et al., “Circ‐HuR Suppresses HuR Expression and Gastric Cancer Progression by Inhibiting CNBP Transactivation,” Molecular Cancer 18, no. 1 (2019): 158. 31718709 10.1186/s12943-019-1094-z PMC6852727

[94] H. Li , W. Jiao , J. Song , et al., “circ‐hnRNPU Inhibits NONO‐mediated c‐myc Transactivation and mRNA Stabilization Essential for Glycosylation and Cancer Progression,” Journal of Experimental & Clinical Cancer Research: CR 42, no. 1 (2023): 313. 37993881 10.1186/s13046-023-02898-5 PMC10666356

[95] L. Wang , H. Long , Q. Zheng , et al., “Circular RNA circRHOT1 Promotes Hepatocellular Carcinoma Progression by Initiation of NR2F6 Expression,” Molecular Cancer 18, no. 1 (2019): 119. 31324186 10.1186/s12943-019-1046-7 PMC6639939

[96] M. Jie , Y. Wu , M. Gao , et al., “CircMRPS35 suppresses Gastric Cancer Progression via Recruiting KAT7 to Govern Histone Modification,” Molecular Cancer 19, no. 1 (2020): 56. 32164722 10.1186/s12943-020-01160-2 PMC7066857

[97] Y. Lin , Y. Wang , L. Li , et al., “Coding Circular RNA in human Cancer,” Genes & Diseases 12, no. 3 (2025): 101347. 40034125 10.1016/j.gendis.2024.101347 PMC11875173

[98] X. Fan , Y. Yang , C. Chen , et al., “Pervasive Translation of Circular RNAs Driven by Short IRES‐Like Elements,” Nature Communications 13, no. 1 (2022): 3751. 10.1038/s41467-022-31327-y PMC9242994 35768398

[99] Y. Zhu , J. Li , H. Yang , et al., “The Potential Role of m6A Reader YTHDF1 as Diagnostic Biomarker and the Signaling Pathways in Tumorigenesis and Metastasis in Pan‐cancer,” Cell Death Discovery 9, no. 1 (2023): 34. 36707507 10.1038/s41420-023-01321-4 PMC9883452

[100] K. Zeng , J. Peng , Y. Xing , et al., “A Positive Feedback Circuit Driven by m6A‐modified Circular RNA Facilitates Colorectal Cancer Liver Metastasis,” Molecular Cancer 22, no. 1 (2023): 202. 38087322 10.1186/s12943-023-01848-1 PMC10717141

[101] R. Song , P. Guo , X. Ren , et al., “A Novel Polypeptide CAPG‐171aa Encoded by circCAPG Plays a Critical Role in Triple‐negative Breast Cancer,” Molecular Cancer 22, no. 1 (2023): 104. 37408008 10.1186/s12943-023-01806-x PMC10320902

[102] X. Yu , P. Ding , M. Guo , et al., “Extracellular Vesicle‐mediated Delivery of circp53 Suppresses the Progression of Multiple Cancers by Activating the CypD/TRAP/HSP90 Pathway,” Experimental & Molecular Medicine 57, no. 8 (2025): 1711–1726. 40744997 10.1038/s12276-025-01506-0 PMC12411616

[103] C. Zhang , C. Tian , R. Zhu , et al., “CircSATB1 promotes Colorectal Cancer Liver Metastasis Through Facilitating FKBP8 Degradation via RNF25‐mediated Ubiquitination,” Advanced Science 12, no. 13 (2025): e2406962. 39921520 10.1002/advs.202406962 PMC11967755

[104] G.‐D. Jia , S.‐Y. Xie , X.‐Y. Li , et al., “Endoplasmic Reticulum‐localized Circular RNA FAM13B Restrains Nasopharyngeal Carcinoma Lymphatic Metastasis Through Downregulating XBP1,” Journal of Experimental & Clinical Cancer Research 44, no. 1 (2025): 223. 40745648 10.1186/s13046-025-03468-7 PMC12312493

[105] Z. Lin , C. Zhong , M. Shi , et al., “Circular RNA TFRC/SCD1 mRNA Interaction Regulates Ferroptosis and Metastasis in Gastric Cancer,” Cell Death & Disease 16, no. 1 (2025): 436. 40473597 10.1038/s41419-025-07759-x PMC12141735

[106] Q. Li , X. Pan , D. Zhu , et al., “Circular RNA MAT2B Promotes Glycolysis and Malignancy of Hepatocellular Carcinoma Through the miR‐338‐3p/PKM2 Axis Under Hypoxic Stress,” Hepatology 70, no. 4 (2019): 1298–1316. 31004447 10.1002/hep.30671

[107] Q. Li , Y. Wang , S. Wu , et al., “CircACC1 regulates Assembly and Activation of AMPK Complex Under Metabolic Stress,” Cell Metabolism 30, no. 1 (2019): 7. 31155494 10.1016/j.cmet.2019.05.009

[108] C. Qian , S. Chen , S. Li , et al., “Circ_0000003 regulates Glutamine Metabolism and Tumor Progression of Tongue Squamous Cell Carcinoma via the miR‑330‑3p/GLS Axis,” Oncology Reports 45, no. 4 (2021): 45. 33649795 10.3892/or.2021.7996 PMC7934215

[109] Y. Cai , Z. Dong , and J. Wang , “Circ_0000808 promotes the Development of Non‐small Cell Lung Cancer by Regulating Glutamine Metabolism via the miR‐1827/SLC1A5 Axis,” World Journal of Surgical Oncology 20, no. 1 (2022): 329. 36192755 10.1186/s12957-022-02777-x PMC9528172

[110] G. Liang , Y. Ling , M. Mehrpour , et al., “Autophagy‐associated circRNA circCDYL Augments Autophagy and Promotes Breast Cancer Progression,” Molecular Cancer 19, no. 1 (2020): 65. 32213200 10.1186/s12943-020-01152-2 PMC7093993

[111] X. Zeng , J. Tang , Q. Zhang , et al., “CircHIPK2 contributes Cell Growth in Intestinal Epithelial of Colitis and Colorectal Cancer Through Promoting TAZ Translation,” Advanced Science 11, no. 34 (2024): e2401588. 38981023 10.1002/advs.202401588 PMC11425914

[112] A. K. Voss and A. Strasser , “The Essentials of Developmental Apoptosis,” F1000Research 9 (2020): 148. 10.12688/f1000research.21571.1 PMC7047912 32148779

[113] A. I. Mahmoud , “Metabolic Switches During Development and Regeneration,” Development (Cambridge, England) 150, no. 20 (2023): dev202008. 37883063 10.1242/dev.202008 PMC10652040

[114] L. Sun , H. Zhang , and P. Gao , “Metabolic Reprogramming and Epigenetic Modifications on the Path to Cancer,” Protein & Cell 13, no. 12 (2022): 877–919. 34050894 10.1007/s13238-021-00846-7 PMC9243210

[115] P. Dey , A. C. Kimmelman , and R. A. DePinho , “Metabolic Codependencies in the Tumor Microenvironment,” Cancer Discovery 11, no. 5 (2021): 1067–1081. 33504580 10.1158/2159-8290.CD-20-1211 PMC8102306

[116] Y. Mo , Y. Wang , S. Zhang , et al., “Circular RNA circRNF13 Inhibits Proliferation and Metastasis of Nasopharyngeal Carcinoma via SUMO2,” Molecular Cancer 20, no. 1 (2021): 112. 34465340 10.1186/s12943-021-01409-4 PMC8406723

[117] Y. Yang , J. Luo , Z. Wang et al., “Energy Stress‐induced circEPB41(2) Promotes Lipogenesis in Hepatocellular Carcinoma,” Cancer Research 85, no. 4 (2025): 723–738. 39636740 10.1158/0008-5472.CAN-24-1630

[118] R. Rui , L. Zhou , and S. He , “Cancer Immunotherapies: Advances and Bottlenecks,” Frontiers in Immunology 14 (2023): 1212476. 37691932 10.3389/fimmu.2023.1212476 PMC10484345

[119] M. Roerden and S. Spranger , “Cancer Immune Evasion, Immunoediting and Intratumour Heterogeneity,” Nature Reviews Immunology 25, no. 5 (2025): 353–369. 10.1038/s41577-024-01111-8 39748116

[120] A. M. Starzer , M. Preusser , and A. S. Berghoff , “Immune Escape Mechanisms and Therapeutic Approaches in Cancer: The Cancer‐immunity Cycle,” Therapeutic Advances in Medical Oncology 14 (2022): 17588359221096219. 35510032 10.1177/17588359221096219 PMC9058458

[121] C. Galassi , T. A. Chan , I. Vitale , et al., “The Hallmarks of Cancer Immune Evasion,” Cancer Cell 42, no. 11 (2024): 1825–1863. 39393356 10.1016/j.ccell.2024.09.010

[122] L. Wang , L. Liang , J. Qian , et al., “Hsa_circ_0007991 promotes Immune Evasion in Hepatocellular Carcinoma via Regulation of the miR‐505‐3p/CANX Axis,” Journal of Hepatocellular Carcinoma 12 (2025): 1337–1351. 40655469 10.2147/JHC.S513120 PMC12254195

[123] Z. Miao , J. Li , Y. Wang , et al., “Hsa_circ_0136666 stimulates Gastric Cancer Progression and Tumor Immune Escape by Regulating the miR‐375/PRKDC Axis and PD‐L1 Phosphorylation,” Molecular Cancer 22, no. 1 (2023): 205. 38093288 10.1186/s12943-023-01883-y PMC10718020

[124] X.‐L. Zhang , L.‐L. Xu , and F. Wang , “Hsa_circ_0020397 regulates Colorectal Cancer Cell Viability, Apoptosis and Invasion by Promoting the Expression of the miR‐138 Targets TERT and PD‐L1,” Cell Biology International 41, no. 9 (2017): 1056–1064. 28707774 10.1002/cbin.10826

[125] S. Gerstberger , Q. Jiang , and K. Ganesh , “Metastasis,” Cell 186, no. 8 (2023): 1564–1579. 37059065 10.1016/j.cell.2023.03.003 PMC10511214

[126] A. Boire , K. Burke , T. R. Cox , et al., “Why Do Patients With Cancer Die?,” Nature Reviews Cancer 24, no. 8 (2024): 578–589. 38898221 10.1038/s41568-024-00708-4 PMC7616303

[127] X. Song , Z. Wei , C. Zhang , et al., “CircAKT3 promotes Prostate Cancer Proliferation and Metastasis by Enhancing the Binding of RPS27A and RPL11,” Molecular Cancer 24, no. 1 (2025): 53. 39994725 10.1186/s12943-025-02261-6 PMC11852832

[128] T. Zhou , Z. Li , W. Huang , et al., “Genome‐wide Analyses Identify circAQR, a Circular RNA Suppressing Proliferation but Promoting Migration and Invasion in Thyroid Cancer,” npj Precision Oncology 9, no. 1 (2025): 171. 40500346 10.1038/s41698-025-00968-9 PMC12159170

[129] X. Li , C. Wang , H. Zhang , et al., “circFNDC3B accelerates Vasculature Formation and Metastasis in Oral Squamous Cell Carcinoma,” Cancer Research 83, no. 9 (2023): 1459–1475. 36811957 10.1158/0008-5472.CAN-22-2585 PMC10152237

[130] C. Mao , M. Wang , L. Zhuang , et al., “Metabolic Cell Death in Cancer: Ferroptosis, Cuproptosis, Disulfidptosis, and Beyond,” Protein & Cell 15, no. 9 (2024): 642–660. 38428031 10.1093/procel/pwae003 PMC11365558

[131] Y. Wang , R. Gao , J. Li , et al., “Circular RNA hsa_circ_0003141 Promotes Tumorigenesis of Hepatocellular Carcinoma via a miR‐1827/UBAP2 Axis,” Aging 12, no. 10 (2020): 9793–9806. 32464601 10.18632/aging.103244 PMC7288939

[132] W. M. D. Vos , H. Tilg , M. Van Hul , and P. D. Cani , “Gut Microbiome and Health: Mechanistic Insights,” Gut 71, no. 5 (2022): 1020–1032. 35105664 10.1136/gutjnl-2021-326789 PMC8995832

[133] Y. Ma , T. Chen , T. Sun , D. Dilimulati , and Y. Xiao , “The Oncomicrobiome: New Insights Into Microorganisms in Cancer,” Microbial Pathogenesis 197 (2024): 107091. 39481695 10.1016/j.micpath.2024.107091

[134] Z. Zhu , J. Huang , X. Li , et al., “Gut Microbiota Regulate Tumor Metastasis via circRNA/miRNA Networks,” Gut Microbes 12, no. 1 (2020): 1788891. 32686598 10.1080/19490976.2020.1788891 PMC7524358

[135] L. Zhang , X. Li , H. Gao , et al., “Gut Microbiota‐lncRNA/circRNA Crosstalk: Implications for Different Diseases,” Critical Reviews in Microbiology 51, no. 3 (2024): 499–513. 38967384 10.1080/1040841X.2024.2375516

[136] Y. Liang , F. Ye , D. Luo , et al., “Exosomal circSIPA1L3‐mediated Intercellular Communication Contributes to Glucose Metabolic Reprogramming and Progression of Triple Negative Breast Cancer,” Molecular Cancer 23, no. 1 (2024): 125. 38849860 10.1186/s12943-024-02037-4 PMC11161950

[137] Y. Sun , B. Han , J. Ge , et al., “CircPSD3 aggravates Tumor Progression by Maintaining TCA Cycle and Mitochondrial Function via Regulating SUCLG2 in Thyroid Carcinoma,” Cell Death & Disease 16, no. 1 (2025): 590. 40925894 10.1038/s41419-025-07856-x PMC12420806

[138] P. Zhao , Z. Zhu , X. Zheng , et al., “Effects of Circulating RNAs on Tumor Metabolism in Lung Cancer (review),” Oncology Letters 29, no. 4 (2025): 1–11. 40070786 10.3892/ol.2025.14950 PMC11894507

[139] J.‐Y. Wu , Y.‐L. Zhou , S.‐H. Lu , et al., “circFOXO3 facilitated Endothelial Cell Senescence and Atherosclerosis Through Binding to HnRNPK,” Genes & Diseases 12, no. 5 (2025): 101517. 40520995 10.1016/j.gendis.2025.101517 PMC12166678

[140] S. Wu , X. Dai , Y. Xia , et al., “Targeting High circDNA2v Levels in Colorectal Cancer Induces Cellular Senescence and Elicits an Anti‐tumor Secretome,” Cell Reports 43, no. 4 (2024): 114111. 38615319 10.1016/j.celrep.2024.114111

[141] A. Q. Yu , Z. X. Wang , W. Wu , K. Y. Chen , and S. R. Yan , “Circular RNA CircCCNB1 Sponges Micro RNA‐449a to Inhibit Cellular Senescence by Targeting CCNE2,” Aging 11, no. 22 (2019): 10220–10241, SR. 31767812 10.18632/aging.102449 PMC6914408

[142] W. W. Du , W. Yang , E. Liu , et al., “Foxo3 circular RNA Retards Cell Cycle Progression via Forming Ternary Complexes With p21 and CDK2,” Nucleic Acids Research 44, no. 6 (2016): 2846–2858. 26861625 10.1093/nar/gkw027 PMC4824104

[143] L. Wang , L. Lankhorst , and R. Bernards , “Exploiting Senescence for the Treatment of Cancer,” Nature Reviews Cancer 22, no. 6 (2022): 340–355. 35241831 10.1038/s41568-022-00450-9

[144] J. Dai , Z. Wang , X. Cheng , et al., “Dynamic Modifications of Circular RNAs Drive Oncogenesis,” Epigenomics 17, no. 11 (2025): 753–762. 40662873 10.1080/17501911.2025.2518918 PMC12320819

[145] N. Chen , G. Zhao , X. Yan , et al., “A Novel FLI1 Exonic Circular RNA Promotes Metastasis in Breast Cancer by Coordinately Regulating TET1 and DNMT1,” Genome Biology 19, no. 1 (2018): 218. 30537986 10.1186/s13059-018-1594-y PMC6290540

[146] Y. Wang , C. Wang , X. Dai , et al., “Deciphering the Multifaceted Role of Circular RNA in Aging: From Molecular Mechanisms to Therapeutic Potentials,” Non‐coding RNA Research 14 (2025): 129–144. 40585063 10.1016/j.ncrna.2025.05.015 PMC12205489

[147] Z. Liu , T. Wang , Y. She , et al., “N6‐methyladenosine‐modified circIGF2BP3 Inhibits CD8+ T‐cell Responses to Facilitate Tumor Immune Evasion by Promoting the Deubiquitination of PD‐L1 in Non‐small Cell Lung Cancer,” Molecular Cancer 20, no. 1 (2021): 105. 34416901 10.1186/s12943-021-01398-4 PMC8377850

[148] S. Huang , Y. Han , Y. Liu , et al., “Super‐enhancer‐mediated circRNAs Exhibit High Splicing Circularization Diversity and Transcriptional Activity,” Nucleic Acids Research 53, no. 11 (2025): gkaf505. 40512546 10.1093/nar/gkaf505 PMC12164585

[149] W. Li , T. Zhao , D. Wu , et al., “Colorectal Cancer in Ulcerative Colitis: Mechanisms, Surveillance and Chemoprevention,” Current Oncology 29, no. 9 (2022): 6091–6114. 36135048 10.3390/curroncol29090479 PMC9498229

[150] C. C. Murphy and T. A. Zaki , “Changing Epidemiology of Colorectal Cancer—birth Cohort Effects and Emerging Risk Factors,” Nature Reviews Gastroenterology & Hepatology 21, no. 1 (2023): 25–34. 37723270 10.1038/s41575-023-00841-9

[151] L. Li , I. Lam , J. Wang , et al., “Epigenetic Mechanism of iPSC‐MSC‐EVs in Colonic Epithelial Cell Pyroptosis in Ulcerative Colitis Cell Models via Modulation of ELF3/miR‐342‐3p/KDM6B Axis and Histone Methylation,” International Immunopharmacology 157 (2025): 114704. 40315630 10.1016/j.intimp.2025.114704

[152] B. Li , Y. Li , L. Li , et al., “Hsa_circ_0001021 regulates Intestinal Epithelial Barrier Function via Sponging miR‐224‐5p in Ulcerative Colitis,” Epigenomics 13, no. 17 (2021): 1385–1401. 34528447 10.2217/epi-2021-0230

[153] Y. Xu , Y. Tian , F. Li , et al., “Circular RNA HECTD1 Mitigates Ulcerative Colitis by Promoting Enterocyte Autophagy via miR‐182‐5p/HuR Axis,” Inflammatory Bowel Diseases 28, no. 2 (2021): 273–288. 10.1093/ibd/izab188 34427642

[154] Y. Yi , Y. Fang , K. Wu , et al., “Comprehensive Gene and Pathway Analysis of Cervical Cancer Progression,” Oncology Letters 19, no. 4 (2020): 3316–3332. 32256826 10.3892/ol.2020.11439 PMC7074609

[155] J. Du , T. Tan , Y. Yu , et al., “The Mechanism of ECT1/E6E7 Cervical Intraepithelial Neoplasia Cells Regulated by acinetobacter lwoffii Through Circ‐LDHA/HMGB1,” BMC Microbiology 25, no. 1 (2025): 326. 40414884 10.1186/s12866-025-04043-y PMC12105123

[156] Y. Zhou , L. Tao , J. Qiu , et al., “Tumor Biomarkers for Diagnosis, Prognosis and Targeted Therapy,” Signal Transduction and Targeted Therapy 9, no. 1 (2024): 132. 38763973 10.1038/s41392-024-01823-2 PMC11102923

[157] L. S. Kristensen , T. Jakobsen , H. Hager , et al., “The Emerging Roles of circRNAs in Cancer and Oncology,” Nature Reviews Clinical Oncology 19, no. 3 (2022): 188–206. 10.1038/s41571-021-00585-y 34912049

[158] M. Perachino C. Ortiz , J. Carmona , et al., “Expanding Screening Through the Use of Liquid Biopsy for Early Cancer Detection,” Communications Medicine 5, no. 1 (2025): 167. 40348826 10.1038/s43856-025-00885-9 PMC12065791

[159] F. Zhang , J. Jiang , H. Qian , et al., “Exosomal circRNA: Emerging Insights Into Cancer Progression and Clinical Application Potential,” Journal of Hematology & Oncology 16, no. 1 (2023): 67. 37365670 10.1186/s13045-023-01452-2 PMC10294326

[160] G. Wen , T. Zhou , and W. Gu , “The Potential of Using Blood Circular RNA as Liquid Biopsy Biomarker for human Diseases,” Protein & Cell 12, no. 12 (2020): 911–946. 33131025 10.1007/s13238-020-00799-3 PMC8674396

[161] X.‐O. Zhang , H.‐B. Wang , Y. Zhang , X. Lu , L.‐L. Chen , and L. Yang , “Complementary Sequence‐mediated Exon Circularization,” Cell 159, no. 1 (2014): 134–147. 25242744 10.1016/j.cell.2014.09.001

[162] Y. Shi and J. Shang , “Circular RNA Expression Profiling by Microarray‐a Technical and Practical Perspective,” Biomolecules 13, no. 4 (2023): 679. 37189426 10.3390/biom13040679 PMC10135611

[163] Y. Gao , J. Wang , and F. Zhao , “CIRI: An Efficient and Unbiased Algorithm for De Novo Circular RNA Identification,” Genome Biology 16, no. 1 (2015): 4. 25583365 10.1186/s13059-014-0571-3 PMC4316645

[164] Z. Zhou , J. Zhang , X. Zheng , et al., “CIRI‐deep Enables Single‐cell and Spatial Transcriptomic Analysis of Circular RNAs With Deep Learning,” Advanced Science 11, no. 14 (2024): e2308115. 38308181 10.1002/advs.202308115 PMC11005702

[165] A. Goytain and T. Ng , “NanoString nCounter Technology: High‐throughput RNA Validation,” Methods in Molecular Biology (Clifton, NJ) 2079 (2019): 125–139. 10.1007/978-1-4939-9904-0_10 31728967

[166] J. M. Eastel , K. W. Lam , N. L. Lee , et al., “Application of NanoString Technologies in Companion Diagnostic Development,” Expert Review of Molecular Diagnostics 19, no. 7 (2019): 591–598. 31164012 10.1080/14737159.2019.1623672

[167] J. Chilimoniuk , A. Erol , S. Rödiger , et al., “Challenges and Opportunities in Processing NanoString nCounter Data,” Computational and Structural Biotechnology Journal 23 (2024): 1951–1958. 38736697 10.1016/j.csbj.2024.04.061 PMC11087919

[168] Z. Mi , C. Zhongqiang , J. Caiyun , et al., “Circular RNA Detection Methods: A Minireview,” Talanta 238, no. Pt 2 (2022): 123066. 34808570 10.1016/j.talanta.2021.123066

[169] K. A. Sillence , L. A. Roberts , H. J. Hollands , et al., “Fetal Sex and RHD Genotyping With Digital PCR Demonstrates Greater Sensitivity Than Real‐time PCR,” Clinical Chemistry 61, no. 11 (2015): 1399–1407. 26354802 10.1373/clinchem.2015.239137

[170] J. F. Huggett , S. Cowen , and C. A. Foy , “Considerations for Digital PCR as an Accurate Molecular Diagnostic Tool,” Clinical Chemistry 61, no. 1 (2015): 79–88. 25338683 10.1373/clinchem.2014.221366

[171] T. Li , Y. Shao , L. Fu , et al., “Plasma Circular RNA Profiling of Patients With Gastric Cancer and Their Droplet Digital RT‐PCR Detection,” Journal of Molecular Medicine 96, no. 1 (2017): 85–96. 29098316 10.1007/s00109-017-1600-y

[172] Z. Zhou , B. Han , Y. Wang , et al., “Fast and Sensitive Multivalent Spatial Pattern‐recognition for Circular RNA Detection,” Nature Communications 15, no. 1 (2024): 10900. 10.1038/s41467-024-55364-x PMC11685481 39738128

[173] Y. Liu , X. Zhang , M. Liu , et al., “Direct Detection of circRNA in Real Samples Using Reverse Transcription‐rolling Circle Amplification,” Analytica Chimica Acta 1101 (2020): 169–175. 32029108 10.1016/j.aca.2019.12.027

[174] J. Dong , Z. Zeng , R. Sun , et al., “Specific and Sensitive Detection of CircRNA Based on Netlike Hybridization Chain Reaction,” Biosensors & Bioelectronics 192 (2021): 113508. 34284304 10.1016/j.bios.2021.113508

[175] M. Boss and C. Arenz , “A Fast and Easy Method for Specific Detection of Circular RNA by Rolling‐circle Amplification,” Chembiochem 21, no. 6 (2020): 793–796. 31584239 10.1002/cbic.201900514 PMC7154740

[176] H. Liu , D. Fang , C. Zhang , et al., “Circular MTHFD2L RNA‐encoded CM‐248aa Inhibits Gastric Cancer Progression by Targeting the SET‐PP2A Interaction,” Molecular Therapy 31, no. 6 (2023): 1739–1755. 37101395 10.1016/j.ymthe.2023.04.013 PMC10277894

[177] Z. Shang , Z. Luo , Y. Wang , et al., “CircHIPK3 contributes to Cisplatin Resistance in Gastric Cancer by Blocking Autophagy‐dependent Ferroptosis,” Journal of Cellular Physiology 238, no. 10 (2023): 2407–2424. 37566605 10.1002/jcp.31093

[178] X. Zhou , G. Yuan , Y. Wu , et al., “EIF4A3‐induced circFIP1L1 Represses miR‐1253 and Promotes Radiosensitivity of Nasopharyngeal Carcinoma,” Cellular and Molecular Life Sciences 79, no. 7 (2022): 357. 35680727 10.1007/s00018-022-04350-x PMC11072984

[179] Y. Zhang , X. Song , Y. Feng , et al., “The circRNA cEMSY Induces Immunogenic Cell Death and Boosts Immunotherapy Efficacy in Lung Adenocarcinoma,” Cancer Research 85, no. 3 (2024): 497–514. 10.1158/0008-5472.CAN-24-1484 PMC11786956 39531509

[180] A. C. Palcau , C. Pulito , V. De Pascale , et al., “CircPVT1 weakens miR‐33a‐5p Unleashing the c‐MYC/GLS1 Metabolic Axis in Breast Cancer,” Journal of Experimental & Clinical Cancer Research 44, no. 1 (2025): 100. 40114244 10.1186/s13046-025-03355-1 PMC11924866

[181] Y. Wu , M. Xu , Z. Feng , et al., “AUF1‐induced Circular RNA hsa_circ_0010467 Promotes Platinum Resistance of Ovarian Cancer Through miR‐637/LIF/STAT3 Axis,” Cellular and Molecular Life Sciences 80, no. 9 (2023): 256. 37589744 10.1007/s00018-023-04906-5 PMC11072515

[182] Y. Zou , A. Yang , B. Chen , et al., “crVDAC3 alleviates Ferroptosis by Impeding HSPB1 Ubiquitination and Confers Trastuzumab Deruxtecan Resistance in HER2‐low Breast Cancer,” Drug Resistance Updates 77 (2024): 101126. 39243601 10.1016/j.drup.2024.101126

[183] A. Du , S. Li , Y. Zhou , et al., “M6A‐mediated Upregulation of circMDK Promotes Tumorigenesis and Acts as a Nanotherapeutic Target in Hepatocellular Carcinoma,” Molecular Cancer 21, no. 1 (2022): 109. 35524319 10.1186/s12943-022-01575-z PMC9074191

[184] E. Song , “A single arm clinical study of dendritic cell vaccine loaded with circular RNA encoding cryptic peptide for patients with HER2‐negative advanced breast cancer.” (2024).

[185] University Hospital, Toulouse. “Deciphering the role of circular RNAs in the pathogenesis and therapy resistance of ALKpositive anaplastic large‐cell lymphoma.” (2023).

[186] S.‐W. Chen , S.‐Q. Zhu , X. Pei , et al., “Cancer Cell‐derived Exosomal circUSP7 Induces CD8+ T Cell Dysfunction and Anti‐PD1 Resistance by Regulating the miR‐934/SHP2 Axis in NSCLC,” Molecular Cancer 20, no. 1 (2021): 144. 34753486 10.1186/s12943-021-01448-x PMC8576933

[187] J. Ge , J. Wang , F. Xiong , et al., “Epstein‐barr Virus‐encoded Circular RNA CircBART2.2 Promotes Immune Escape of Nasopharyngeal Carcinoma by Regulating PD‐L1,” Cancer Research 81, no. 19 (2021): 5074–5088. 34321242 10.1158/0008-5472.CAN-20-4321 PMC8974435

[188] Y.‐P. Xu , Z.‐N. Dong , S.‐W. Wang , et al., “circHMGCS1‐016 reshapes Immune Environment by Sponging miR‐1236‐3p to Regulate CD73 and GAL‐8 Expression in Intrahepatic Cholangiocarcinoma,” Journal of Experimental & Clinical Cancer Research 40, no. 1 (2021): 290. 34526098 10.1186/s13046-021-02095-2 PMC8442376

[189] Y. Wang , R. Gao , J. Li , et al., “Downregulation of hsa_circ_0074854 Suppresses the Migration and Invasion in Hepatocellular Carcinoma via Interacting With HuR and via Suppressing Exosomes‐mediated Macrophage M2 Polarization,” International Journal of Nanomedicine 16 (2021): 2803–2818. 33880025 10.2147/IJN.S284560 PMC8052130

[190] Y. Wang , Y. Cui , X. Li , et al., “CircRNAs: Functions and Emerging Roles in Cancer and Immunotherapy,” BMC Medicine 23, no. 1 (2025): 477. 40817061 10.1186/s12916-025-04306-5 PMC12357350

[191] R. Woodruff , F. Parekh , K. Lamb , et al., “Large‐scale Manufacturing of Base‐edited Chimeric Antigen Receptor T Cells,” Molecular Therapy Methods & Clinical Development 31 (2023): 101123. 37886606 10.1016/j.omtm.2023.101123 PMC10597784

[192] J. Cai , S. Chen , Z. Liu , et al., “RNA Technology and Nanocarriers Empowering in Vivo Chimeric Antigen Receptor Therapy,” Immunology 173, no. 4 (2024): 634–653. 39340367 10.1111/imm.13861

[193] Z. Li , S. Yin , K. Yang , et al., “CircRNA Regulation of T Cells in Cancer: Unraveling Potential Targets,” International Journal of Molecular Sciences 25, no. 12 (2024): 6383. 38928088 10.3390/ijms25126383 PMC11204142

[194] S. Huang , J. Xu , N. Baran , et al., “Advancing the next Generation of Cancer Treatment With Circular RNAs in CAR‐T Cell Therapy,” Biomedicine & Pharmacotherapy 181 (2024): 117753. 39667221 10.1016/j.biopha.2024.117753

[195] Y. Wang , L. Lin , X. Wang , et al., “Synergically Enhanced Anti‐tumor Immunity of in Vivo panCAR by circRNA Vaccine Boosting,” Cell Reports Medicine 6, no. 8 (2025): 102250. 40712575 10.1016/j.xcrm.2025.102250 PMC12432353

[196] Y. Ma , T. Wang , X. Zhang , et al., “The Role of Circular RNAs in Regulating Resistance to Cancer Immunotherapy: Mechanisms and Implications,” Cell Death & Disease 15, no. 5 (2024): 312. 38697964 10.1038/s41419-024-06698-3 PMC11066075

[197] C. Zhou , W. Li , Z. Liang , et al., “Mutant KRAS‐activated circATXN7 Fosters Tumor Immunoescape by Sensitizing Tumor‐specific T Cells to Activation‐induced Cell Death,” Nature Communications 15, no. 1 (2024): 499. 10.1038/s41467-024-44779-1 PMC10786880 38216551

[198] H. Li , Y. Hu , J. Li , et al., “Intranasal Prime‐boost RNA Vaccination Elicits Potent T Cell Response for Lung Cancer Therapy,” Signal Transduction and Targeted Therapy 10, no. 1 (2025): 101. 40122855 10.1038/s41392-025-02191-1 PMC11930932

[199] H. Li , K. Peng , K. Yang , et al., “Circular RNA Cancer Vaccines Drive Immunity in Hard‐to‐treat Malignancies,” Theranostics 12, no. 14 (2022): 6422–6436. 36168634 10.7150/thno.77350 PMC9475446

[200] L. Ma , A. Hostetler , D. M. Morgan , et al., “Vaccine‐boosted CAR T Crosstalk With Host Immunity to Reject Tumors With Antigen Heterogeneity,” Cell 186, no. 15 (2023): 3148–3165. 37413990 10.1016/j.cell.2023.06.002 PMC10372881

[201] M. Alahdal and E. Elkord , “Non‐coding RNAs in Cancer Immunotherapy: Predictive Biomarkers and Targets,” Clinical and Translational Medicine 13, no. 9 (2023): e1425. 37735815 10.1002/ctm2.1425 PMC10514379

[202] X. Liu , S. Wang , Y. Sun , et al., “Unlocking the Potential of Circular RNA Vaccines: A Bioinformatics and Computational Biology Perspective,” eBioMedicine 114 (2025): 105638. 40112741 10.1016/j.ebiom.2025.105638 PMC11979485

[203] J. Dong , Z. Zeng , Y. Huang , et al., “Challenges and Opportunities for circRNA Identification and Delivery,” Critical Reviews in Biochemistry and Molecular Biology 58, no. 1 (2023): 19–35. 36916323 10.1080/10409238.2023.2185764

[204] Y. G. Chen , M. V. Kim , X. Chen , et al., “Sensing Self and Foreign Circular RNAs by Intron Identity,” Molecular Cell 67, no. 2 (2017): 228–238. 28625551 10.1016/j.molcel.2017.05.022 PMC5610545

[205] R. A. Wesselhoeft , P. S. Kowalski , F. C. Parker‐Hale , et al., “RNA Circularization Diminishes Immunogenicity and Can Extend Translation Duration in Vivo,” Molecular Cell 74, no. 3 (2019): 508–520. e4. 30902547 10.1016/j.molcel.2019.02.015 PMC6724735

[206] Y. Zong , Y. Lin , T. Wei , et al., “Lipid Nanoparticle (LNP) Enables mRNA Delivery for Cancer Therapy,” Advanced Materials 35, no. 51 (2023): e2303261. 37196221 10.1002/adma.202303261

[207] M. Estapé Senti , L. García Del Valle , and R. M. Schiffelers , “mRNA Delivery Systems for Cancer Immunotherapy: Lipid Nanoparticles and Beyond,” Advanced Drug Delivery Reviews 206 (2024): 115190. 38307296 10.1016/j.addr.2024.115190

[208] S. Xu , Y. Xu , N. C. Solek , et al., “Tumor‐tailored Ionizable Lipid Nanoparticles Facilitate IL‐12 Circular RNA Delivery for Enhanced Lung Cancer Immunotherapy,” Advanced Materials 36, no. 29 (2024): e2400307. 38657273 10.1002/adma.202400307

[209] T. J. Smith , Z. C. Elmore , R. M. Fusco , et al., “Engineered IgM and IgG Cleaving Enzymes for Mitigating Antibody Neutralization and Complement Activation in AAV Gene Transfer,” Molecular Therapy 32, no. 7 (2024): 2080–2093. 38715362 10.1016/j.ymthe.2024.05.004 PMC11286816

[210] Z. Xu , S. Zeng , Z. Gong , et al., “Exosome‐based Immunotherapy: A Promising Approach for Cancer Treatment,” Molecular Cancer 19, no. 1 (2020): 160. 33183286 10.1186/s12943-020-01278-3 PMC7661275

[211] J. Shao , J. Zaro , and Y. Shen , “Advances in Exosome‐based Drug Delivery and Tumor Targeting: From Tissue Distribution to Intracellular Fate,” International Journal of Nanomedicine 15 (2020): 9355–9371. 33262592 10.2147/IJN.S281890 PMC7700079

[212] X. Wang , H. Zhang , H. Yang , et al., “Exosome‐delivered circRNA Promotes Glycolysis to Induce Chemoresistance Through the miR‐122‐PKM2 Axis in Colorectal Cancer,” Molecular Oncology 14, no. 3 (2020): 539–555. 31901148 10.1002/1878-0261.12629 PMC7053238

[213] J. Cai and Z. Qiu , “Synthetic circRNA Therapeutics: Innovations, Strategies, and Future Horizons,” MedComm 5, no. 11 (2024): e720. 39525953 10.1002/mco2.720 PMC11550093

[214] C.‐I. Su , Z.‐S. Chuang , C.‐T. Shie , et al., “A Cis‐acting Ligase Ribozyme Generates Circular RNA in Vitro for Ectopic Protein Functioning,” Nature Communications 15, no. 1 (2024): 6607. 10.1038/s41467-024-51044-y PMC11298514 39098891

[215] L. Wang , C. Dong , W. Zhang , et al., “Developing an Enhanced Chimeric Permuted Intron‐exon System for Circular RNA Therapeutics,” Theranostics 14, no. 15 (2024): 5869–5882. 39346546 10.7150/thno.98214 PMC11426236

[216] Y. Du , P. K. Zuber , H. Xiao , X. Li , Y. Gordiyenko , and V. Ramakrishnan , “Efficient Circular RNA Synthesis for Potent Rolling Circle Translation,” Nature Biomedical Engineering 9, no. 7 (2025): 1062–1074. 10.1038/s41551-024-01306-3 PMC12270912 39672985

[217] Y. Shen , B. Li , L. Dong , et al., “Self‐splicing RNA Circularization Facilitated by Intact Group I and II Introns,” Nature Communications 16, no. 1 (2025): 7376. 10.1038/s41467-025-62607-y PMC12336344 40784880

[218] Z. He , H. Ji , B. Xia , et al., “Invention of circRNA Promoting RNA to Specifically Promote circRNA Production,” Nucleic Acids Research 52, no. 17 (2024): e83–e83. 39119897 10.1093/nar/gkae693 PMC11417354

[219] M. J. Unti and S. R. Jaffrey , “Highly Efficient Cellular Expression of Circular mRNA Enables Prolonged Protein Expression,” Cell Chemical Biology 31, no. 1 (2024): 163–176. e5. 37883972 10.1016/j.chembiol.2023.09.015 PMC10841545

[220] M. Tong , N. Palmer , A. Dailamy , et al., “Robust Genome and Cell Engineering via in Vitro and in Situ Circularized RNAs,” Nature Biomedical Engineering 9, no. 1 (2025): 109–126. 10.1038/s41551-024-01245-z PMC12186994 39187662

[221] R. Chen , S. K. Wang , J. A. Belk , et al., “Engineering Circular RNA for Enhanced Protein Production,” Nature Biotechnology 41, no. 2 (2023): 262–272. 10.1038/s41587-022-01393-0 PMC9931579 35851375

[222] Z. Zhang , W. Li , X. Ren , et al., “Mitigating Cellular Dysfunction Through Contaminant Reduction in Synthetic circRNA for High‐efficiency mRNA‐based Cell Reprogramming,” Advanced Science 12, no. 16 (2025): e2416629. 40042035 10.1002/advs.202416629 PMC12021033

[223] N. M. Bemben and M. L. Berg , “Efficacy of Inactivated Vaccines in Patients Treated With Immunosuppressive Drug Therapy,” Pharmacotherapy: The Journal of Human Pharmacology and Drug Therapy 42, no. 4 (2022): 334–342. 10.1002/phar.2671 PMC9088666 35146780

[224] Y. Zhang , G. Zeng , H. Pan , et al., “Safety, Tolerability, and Immunogenicity of an Inactivated SARS‐CoV‐2 Vaccine in Healthy Adults Aged 18–59 Years: A Randomised, Double‐blind, Placebo‐controlled, Phase 1/2 Clinical Trial,” The Lancet Infectious Diseases 21, no. 2 (2021): 181–192. 33217362 10.1016/S1473-3099(20)30843-4 PMC7832443

[225] J. Yu , N. D. Collins , N. B. Mercado , et al., “Protective Efficacy of Gastrointestinal SARS‐CoV‐2 Delivery Against Intranasal and Intratracheal SARS‐CoV‐2 Challenge in rhesus Macaques,” Journal of Virology 96, no. 2 (2022): e0159921. 34705557 10.1128/JVI.01599-21 PMC8791250

[226] S. Pagliari , B. Dema , A. Sanchez‐Martinez , G. Montalvo Zurbia‐Flores , and C. S. Rollier , “DNA Vaccines: History, Molecular Mechanisms and Future Perspectives,” Journal of Molecular Biology 435, no. 23 (2023): 168297. 37797831 10.1016/j.jmb.2023.168297

[227] U. Sahin , K. Karikó , and Ö. Türeci , “mRNA‐based Therapeutics–developing a New Class of Drugs,” Nature Reviews Drug Discovery 13, no. 10 (2014): 759–780. 25233993 10.1038/nrd4278

[228] T. E. Mulroney , T. Pöyry , J. C. Yam‐Puc , et al., “N1‐methylpseudouridylation of mRNA Causes +1 Ribosomal Frameshifting,” Nature 625, no. 7993 (2023): 189–194. 38057663 10.1038/s41586-023-06800-3 PMC10764286

[229] F. Wang , G. Cai , Y. Wang , et al., “Circular RNA‐based Neoantigen Vaccine for Hepatocellular Carcinoma Immunotherapy,” MedComm 5, no. 8 (2024): e667. 39081513 10.1002/mco2.667 PMC11286538

[230] Y. Zhang , W. Wang , X. Qian , et al., “Programmable Aptamer‐embedded Circular rnas for Targeted Antigen‐presenting Cells Immunotherapy,” bioRxiv : The Preprint Server for Biology (2025).

[231] W. Wu , J. Zhang , X. Cao , et al., “Exploring the Cellular Landscape of Circular RNAs Using Full‐length Single‐cell RNA Sequencing,” Nature Communications 13, no. 1 (2022): 3242. 10.1038/s41467-022-30963-8 PMC9187688 35688820

[232] J. Feng , W. Chen , X. Dong , et al., “CSCD2: An Integrated Interactional Database of Cancer‐specific Circular RNAs,” Nucleic Acids Research 50, no. D1 (2021): D1179–D1183. 10.1093/nar/gkab830 PMC8728299 34551437

[233] S. Wang , Y. Xiong , Y. Zhang , et al., “TCCIA: A Comprehensive Resource for Exploring CircRNA in Cancer Immunotherapy,” Journal for ImmunoTherapy of Cancer 12, no. 1 (2024): e008040. 38212124 10.1136/jitc-2023-008040 PMC10806567

[234] J. N. Vo , M. Cieslik , Y. Zhang , et al., “The Landscape of Circular RNA in Cancer,” Cell 176, no. 4 (2019): 869–881. 30735636 10.1016/j.cell.2018.12.021 PMC6601354

[235] X. Chen , P. Han , T. Zhou , et al., “circRNADb: A Comprehensive Database for human Circular RNAs With Protein‐coding Annotations,” Scientific Reports 6, no. 1 (2016): 34985. 27725737 10.1038/srep34985 PMC5057092

[236] Z.‐Y. Sun , C.‐L. Yang , L.‐J. Huang , et al., “circRNADisease v2.0: An Updated Resource for High‐quality Experimentally Supported circRNA‐disease Associations,” Nucleic Acids Research 52, no. D1 (2023): D1193–D1200. 10.1093/nar/gkad949 PMC10767896 37897359

[237] W. S. Alharbi and M. Rashid , “A Review of Deep Learning Applications in human Genomics Using next‐generation Sequencing Data,” Human Genomics 16, no. 1 (2022): 26. 35879805 10.1186/s40246-022-00396-x PMC9317091

[238] C. Khunsriraksakul , D. McGuire , R. Sauteraud , et al., “Integrating 3D Genomic and Epigenomic Data to Enhance Target Gene Discovery and Drug Repurposing in Transcriptome‐wide Association Studies,” Nature Communications 13, no. 1 (2022): 3258. 10.1038/s41467-022-30956-7 PMC9171100 35672318

[239] A. Vefghi , Z. Rahmati , and M. Akbari , “Drug‐target Interaction/Affinity Prediction: Deep Learning Models and Advances Review,” Computers in Biology and Medicine 196, no. Pt A (2025): 110438. 40609289 10.1016/j.compbiomed.2025.110438

[240] P. Ji , W. Wu , S. Chen , et al., “Expanded Expression Landscape and Prioritization of Circular RNAs in Mammals,” Cell Reports 26, no. 12 (2019): 3444–3460. e5. 30893614 10.1016/j.celrep.2019.02.078

[241] Y. Gao and F. Zhao , “Computational Strategies for Exploring Circular RNAs,” Trends in Genetics 34, no. 5 (2018): 389–400. 29338875 10.1016/j.tig.2017.12.016

[242] L. Wang , L. Wong , Z. Li , et al., “A Machine Learning Framework Based on Multi‐source Feature Fusion for circRNA‐disease Association Prediction,” Briefings in Bioinformatics 23, no. 5 (2022): bbac388. 36070867 10.1093/bib/bbac388

[243] C. Lu , L. Zhang , M. Zeng , et al., “Inferring Disease‐associated circRNAs by Multi‐source Aggregation Based on Heterogeneous Graph Neural Network,” Briefings in Bioinformatics 24, no. 1 (2022): bbac549. 10.1093/bib/bbac549 36572658

[244] C. Lu , M. Zeng , F.‐X. Wu , et al., “Improving circRNA‐disease Association Prediction by Sequence and Ontology Representations With Convolutional and Recurrent Neural Networks,” Bioinformatics 36, no. 24 (2020): 5656–5664. 10.1093/bioinformatics/btaa1077 33367690

[245] Y. Zhao , X. He , J. Shang , et al., “AGDFCDA: Adaptive Graph Convolutional Network and Dual Feature for circRNA‐disease Association Prediction,” Journal of Computational Science 90 (2025): 102615.

[246] R. A. Wesselhoeft , P. S. Kowalski , and D. G. Anderson , “Engineering Circular RNA for Potent and Stable Translation in Eukaryotic Cells,” Nature Communications 9, no. 1 (2018): 2629. 10.1038/s41467-018-05096-6 PMC6035260 29980667

[247] C. Xu , C. Pu , R. Chen , et al., “circDesign Algorithm for Designing Synthetic Circular RNA,” bioRxiv : The Preprint Server for Biology (2023).

[248] M. Ramanathan , D. F. Porter , and P. A. Khavari , “Methods to Study RNA‐protein Interactions,” Nature Methods 16, no. 3 (2019): 225–234. 30804549 10.1038/s41592-019-0330-1 PMC6692137

[249] Y. Janapala , K. Woodward , J. Lee , et al., “Rapid in Vivo Fixation and Isolation of Translational Complexes From Eukaryotic Cells,” Journal of Visualized Experiments no. 178 (2021). 10.3791/62639 35001907

[250] X. Dong , K. Chen , W. Chen , et al., “circRIP: An Accurate Tool for Identifying circRNA‐RBP Interactions,” Briefings in Bioinformatics 23, no. 4 (2022): bbac186. 35641157 10.1093/bib/bbac186

[251] Z. Wang , X. Lei , and F.‐X. Wu , “Identifying Cancer‐specific circRNA‐RBP Binding Sites Based on Deep Learning,” Molecules (Basel, Switzerland) 24, no. 22 (2019): 4035. 31703384 10.3390/molecules24224035 PMC6891306

[252] Y. Yang , Z. Hou , Y. Wang , et al., “HCRNet: High‐throughput circRNA‐binding Event Identification From CLIP‐seq Data Using Deep Temporal Convolutional Network,” Briefings in Bioinformatics 23, no. 2 (2022). 10.1093/bib/bbac027 35189638

[253] D. Lasantha , S. Vidanagamachchi , and S. Nallaperuma , “CRIECNN: Ensemble Convolutional Neural Network and Advanced Feature Extraction Methods for the Precise Forecasting of circRNA‐RBP Binding Sites,” Computers in Biology and Medicine 174 (2024): 108466. 38615462 10.1016/j.compbiomed.2024.108466

[254] R. Han , X. Liu , T. Pan , et al., “CoPRA: Bridging Cross‐domain Pretrained Sequence Models With Complex Structures for Protein‐RNA Binding Affinity Prediction,” Proceedings of the AAAI Conference on Artificial Intelligence 39, no. 1 (2025): 246–254.

[255] A. Dance , “Circular Logic: Understanding RNA's Strangest Form yet,” Nature 635, no. 8038 (2024): 511–513. 39528868 10.1038/d41586-024-03683-w

[256] M. Vromman , J. Anckaert , S. Bortoluzzi , et al., “Large‐scale Benchmarking of circRNA Detection Tools Reveals Large Differences in Sensitivity but Not in Precision,” Nature Methods 20, no. 8 (2023): 1159–1169. 37443337 10.1038/s41592-023-01944-6 PMC10870000

[257] T. E. Mullen and W. F. Marzluff , “Degradation of Histone mRNA Requires Oligouridylation Followed by Decapping and Simultaneous Degradation of the mRNA both 5' to 3' and 3' to 5',” Genes & Development 22, no. 1 (2008): 50–65. 18172165 10.1101/gad.1622708 PMC2151014

[258] E. Gaffo , A. Buratin , A. Dal Molin , and S. Bortoluzzi , “Sensitive, Reliable and Robust circRNA Detection From RNA‐seq With CirComPara2,” Briefings in Bioinformatics 23, no. 1 (2021): bbab418. 10.1093/bib/bbab418 PMC8769706 34698333

[259] S. K. Bowman , M. D. Simon , A. M. Deaton , et al., “Multiplexed illumina Sequencing Libraries From Picogram Quantities of DNA,” BMC Genomics [Electronic Resource] 14, no. 1 (2013): 466. 23837789 10.1186/1471-2164-14-466 PMC3711846

[260] S. Huang , W. Shi , S. Li , et al., “Advanced Sequencing‐based High‐throughput and Long‐read Single‐cell Transcriptome Analysis,” Lab on a Chip 24, no. 10 (2024): 2601–2621. 38669201 10.1039/d4lc00105b

[261] J. Zhang and F. Zhao , “Circular RNA Discovery With Emerging Sequencing and Deep Learning Technologies,” Nature Genetics 57, no. 5 (2025): 1089–1102. 40247051 10.1038/s41588-025-02157-7

[262] Y. Fu , H. Kim , S. Roy , et al., “Single Cell and Spatial Alternative Splicing Analysis With Nanopore Long Read Sequencing,” Nature Communications 16, no. 1 (2025): 6654. 10.1038/s41467-025-60902-2 PMC12276307 40683866

[263] M. Cao , G.‐H. Yuan , S.‐M. Cao , et al., “Direct circMAN1A2(2,3,4,5)‐CENPB mRNA Interaction Regulates Cell Proliferation and Cancer Progression,” Nature Communications 16, no. 1 (2025): 8609. 10.1038/s41467-025-63686-7 PMC12480695 41022764

[264] E. Hutchins , R. Reiman , J. Winarta , et al., “Extracellular Circular RNA Profiles in Plasma and Urine of Healthy, Male College Athletes,” Scientific Data 8, no. 1 (2021): 276. 34711851 10.1038/s41597-021-01056-w PMC8553830

[265] X. Wei , Y. Shi , Z. Dai , et al., “Underlying Metastasis Mechanism and Clinical Application of Exosomal Circular RNA in Tumors (review),” International Journal of Oncology 58, no. 3 (2021): 289–297. 33650643 10.3892/ijo.2021.5179 PMC7864150

[266] D. Pullman and H. Etchegary , “Ethical, Legal, and Social Issues (ELSI) in Clinical Genetics Research,” Methods in Molecular Biology (Clifton, NJ) 2249 (2021): 65–82. 10.1007/978-1-0716-1138-8_5 33871839

[267] D. G. Morrison , C. Farah , and J. M. Hock , “Informed Consent for Biobanking Research: Cancer Patient Recruitment From Rural Communities in maine,” Biopreservation and Biobanking 11, no. 2 (2013): 107–114. 24845431 10.1089/bio.2012.0054

[268] H. J. A. Teare , M. Prictor , and J. Kaye , “Reflections on Dynamic Consent in Biomedical Research: The Story so Far,” European Journal of Human Genetics 29, no. 4 (2020): 649–656. 33249421 10.1038/s41431-020-00771-z PMC7695991

[269] L. Riva and C. Petrini , “A Few Ethical Issues in Translational Research for Gene and Cell Therapy,” Journal of Translational Medicine 17, no. 1 (2019): 395. 31779636 10.1186/s12967-019-02154-5 PMC6883654

[270] J. Luo , S. Ou , H. Wei , et al., “Value Assessment of NMPA‐approved New Cancer Drugs for Solid Cancer in China, 2016–2020,” Frontiers in Public Health 11 (2023): 1109668. 36908440 10.3389/fpubh.2023.1109668 PMC9998930

[271] G. Cossu , M. Birchall , T. Brown , et al., “Lancet Commission: Stem Cells and Regenerative Medicine,” The Lancet 391, no. 10123 (2018): 883–910. 10.1016/S0140-6736(17)31366-1 28987452

[272] B. R. Giri and G. Cheng , “A Review on Circular RNAs in Helminth Parasites: Insights and Potential Applications,” International Journal of Biological Macromolecules 333, no. Pt 2 (2025): 148956. 41232869 10.1016/j.ijbiomac.2025.148956

[273] Y. Rahmati , Y. Asemani , S. Aghamiri , et al., “CiRS‐7/CDR1as; an Oncogenic Circular RNA as a Potential Cancer Biomarker,” Pathology—Research and Practice 227 (2021): 153639. 34649055 10.1016/j.prp.2021.153639

[274] S. T. Hess , T. P. K. Girirajan , and M. D. Mason , “Ultra‐high Resolution Imaging by Fluorescence Photoactivation Localization Microscopy,” Biophysical Journal 91, no. 11 (2006): 4258–4272. 16980368 10.1529/biophysj.106.091116 PMC1635685

[275] S.‐M. Cao , H. Wu , G.‐H. Yuan , et al., “Altered Nucleocytoplasmic Export of Adenosine‐rich circRNAs by PABPC1 Contributes to Neuronal Function,” Molecular Cell 84, no. 12 (2024): 2304–2319. e8. 38838666 10.1016/j.molcel.2024.05.011

[276] C.‐X. Liu , S.‐K. Guo , F. Nan , Y.‐F. Xu , L. Yang , and L.‐L. Chen , “RNA Circles With Minimized Immunogenicity as Potent PKR Inhibitors,” Molecular Cell 82, no. 2 (2022): 420–434. 34951963 10.1016/j.molcel.2021.11.019

[277] T. Loan Young , K. Chang Wang , A. James Varley , and B. Li , “Clinical Delivery of Circular RNA: Lessons Learned From RNA Drug Development,” Advanced Drug Delivery Reviews 197 (2023): 114826. 37088404 10.1016/j.addr.2023.114826

[278] M. Hosseini‐Kharat , K. E. Bremmell , and C. A. Prestidge , “Why Do Lipid Nanoparticles Target the Liver? Understanding of Biodistribution and Liver‐specific Tropism,” Molecular Therapy Methods & Clinical Development 33, no. 1 (2025): 101436. 40104152 10.1016/j.omtm.2025.101436 PMC11919328

[279] Y. Zhao and H. Wang , “Artificial Intelligence‐driven circRNA Vaccine Development: Multimodal Collaborative Optimization and a New Paradigm for Biomedical Applications,” Briefings in Bioinformatics 26, no. 3 (2025): bbaf263. 40483546 10.1093/bib/bbaf263 PMC12145227

